# Tungsten-Embedded Graphene: Theoretical Study on a Potential High-Activity Catalyst toward CO Oxidation

**DOI:** 10.3390/ma11101848

**Published:** 2018-09-28

**Authors:** Guoliang Dai, Lei Chen, Xin Zhao

**Affiliations:** Jiangsu Key Laboratory for Environment Functional Materials, School of Chemistry Biology and Material Engineering, Suzhou University of Science and Technology, Suzhou 215009, China; 1713091007@post.usts.edu.cn (L.C.); zhaoxin_sz@mail.usts.edu.cn (X.Z.)

**Keywords:** tungsten, natural bond orbital (NBO), CO oxidation, graphene

## Abstract

The oxidation mechanism of CO on W-embedded graphene was investigated by M06-2X density functional theory. Two models of tungsten atom embedded in single and double vacancy (W-SV and W-DV) graphene sheets were considered. It was found that over W-SV-graphene and W-DV-graphene, the oxidation of CO prefers to Langmuir-Hinshelwood (LH) and Eley-Rideal (ER) mechanism, respectively. The two surfaces exhibit different catalytic activity during different reaction stages. The present results imply that W-embedded graphene is a promising catalyst for CO oxidation, which provides a useful reference for the design of a high-efficiency catalyst in detecting and removing of toxic gases.

## 1. Introduction 

Environmental pollution caused by automobile and industrial emissions has become a serious threat to human health and ecological safety; carbon monoxide is one of the main pollutants in these exhaust gases. It is well known that catalytic oxidation of carbon monoxide with noble metals such as Au, Pt, and Pd as catalysts is an effective method to eliminate this hazardous gas, but the high cost will undoubtedly limit their widespread use [1,2]. Therefore, it is particularly important to develop low-cost catalysts with high-catalytic activity for carbon monoxide oxidation. As a two-dimensional nanomaterial, graphene is used as an ideal catalytic support in many fields due to its excellent electric conductivity and large surface. For example, the nitrogen-doped graphene was demonstrated to act as a metal-free electrode with an excellent electrocatalytic activity for oxygen reduction in alkaline fuel cells [3]. Recent research suggests graphene oxide (GO) can enhance photocatalytic activity of some catalysts [4], and it is a potential advanced membrane material for desalination and gas separation [5]. Moreover, it is found that transition metal-embedded graphene exhibits excellent catalytic performance for carbon monoxide oxidation. Not only some noble metals such as gold, palladium, platinum, and ruthenium [6,7,8,9], but also many common metals such as iron, nickel, and copper [10,11,12,13,14,15,16,17], and even some non-metals such as silicon, germanium and boron-embedded graphene [18,19,20,21] exhibit superior catalytic activity toward CO oxidation at low temperatures. In the past several years, many theoretical studies have evaluated the performance of some impurity atoms-embedded graphene toward CO oxidation, and proposed some feasible catalytic mechanisms such as Langmuir-Hinshelwood (LH) and Eley-Rideal (ER) mechanisms, which can provide useful guidance for the design of high-efficiency catalysts in the future. It is generally believed that graphene has significant adsorptive power for certain gases [22] and solvents molecules [23], and during the process of CO oxidation reaction, the metal atom site often acts as an active center on the surface of metal-embedded graphene, which plays a key role in the adsorbing of gas molecules, activating oxygen, and then oxidizing carbon monoxide. The ability of metal atoms to adsorb and activate oxygen is the key factor in determining the oxidation mechanism. In fact, the catalytic oxidation mechanism of carbon monoxide on metal-embedded graphene is complicated. Besides the embedded impurity atom, the oxygen atom formed on the surface during the reaction may also become the active center for the CO oxidation. Previous studies have proved that a variety of oxygen species can be formed on a different site of the catalyst surface during the whole reaction process. However, there is no sufficient understanding of the reactivity of different oxygen species to date. Furthermore, it is found that previous theoretical studies mainly focus on the model of metal atoms embedded in single vacancy (SV) in graphene sheets. However, recent experimental studies have confirmed that both single vacancy (SV) and double vacancy (DV) can exist simultaneously on the surface of graphene [24]. Theoretically, the structures of transition metal atoms adsorbed on defective graphene (including SV and DV) were calculated by Krasheninnnikov et al. [25], they found in both cases the metal atom can bond strongly with surface, and the diffusion barrier of metal atoms is high, indicating the metal-embedded graphene can be utilized in catalytic fields due to its strong stability. Besides, previous studies focused on graphene embedded with 3*d* and 4*d* transition metals, only noble metals platinum and gold were explored for the 5*d* metals. As a common 5*d* transition metal in the sixth period, the supported tungsten and its oxides by graphene are widely used in many fields of photocatalyst, antibacterial, anticancer agent, and gas sensors [26,27,28]. Recent research indicates this composite exhibit high selective catalytic reduction (SCR) efficiency even at high temperatures [29]. Moreover, in the hydrogen evolution reaction, nano-tungsten carbide decorated graphene also shows an enhanced catalytic performance [30]. Theoretically, the magnetic behaviors and electronic structures of graphene with tungsten doped were investigated by Luan and Tang et al. [31,32]. The results indicated the tungsten was bonded to single vacancy tightly to form a stable substitution system, they believe this embedded graphene can be used in nano electronics, spintronics, and magnetic storage devices. Jin et al. [33] have investigated the mechanism of CO oxidation on the WO_3_(001) surface systematically, their calculation results show that this tungsten oxide is one of the most promising gas sensor candidates due to its high activity toward CO oxidation. To the best of our knowledge, the mechanism of CO oxidation on tungsten-embedded graphene is still lacking in experimental and theoretical investigations. Does this composite also exhibit excellent activity for CO oxidation? What kind of mechanisms are adopted in the reaction process? In order to understand the related mechanism more deeply, in the present work, the tungsten-embedded graphene was taken as the computational model, the related oxidation mechanism was investigated through theoretical calculation. The purpose of this study is not only to evaluate the activity of this catalyst, but also to reveal the microscopic mechanism of this kind of reaction, and to understand the activity of different oxygen species and different types of vacancies (i.e., SV and DV) during the reaction process. 

## 2. Computational Details

In the present work, the density functional theory (DFT) method is employed to study the detailed reaction mechanism at the molecular level. The geometry optimization and the subsequent frequency analysis of the complexes involved in the reaction were performed using the M06-2X density functional, all the calculations were performed using the Gaussian 09 package [34,35]. In the DFT calculations, the Stuttgart/Dresden ECPs (SDD) basis set was used for tungsten, and the standardized 6-31G* basis set was used for non-metallic atoms. A hexagonal super cell (4 × 4 unit cell) containing forty-eight carbon atoms was chosen as the computational model for the present study. Esrafili et al. [21] used a similar model for theoretical research and achieved satisfactory results before. The W-embedded graphene was simulated by replacing one or two carbon atoms with a single tungsten atom on the surface, named W-SV-graphene or W-DV-graphene. All the calculations were carried out in gas phase. The adsorption energy (E*_ads_*) was calculated as Equation (1):

E*_ads_* = E(*_adsorbate-substrate_*) − (E*_adsorbate_* + E*_substrate_*) (1)

## 3. Results and Discussion 

### 3.1. Tungsten-Embedded Graphene

**Figure 1:**
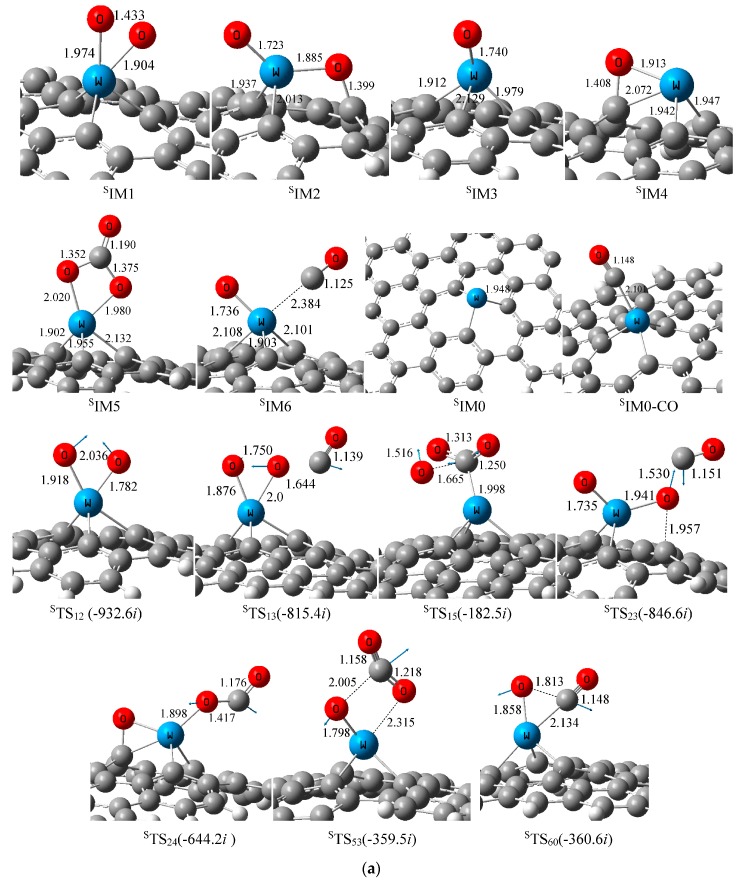

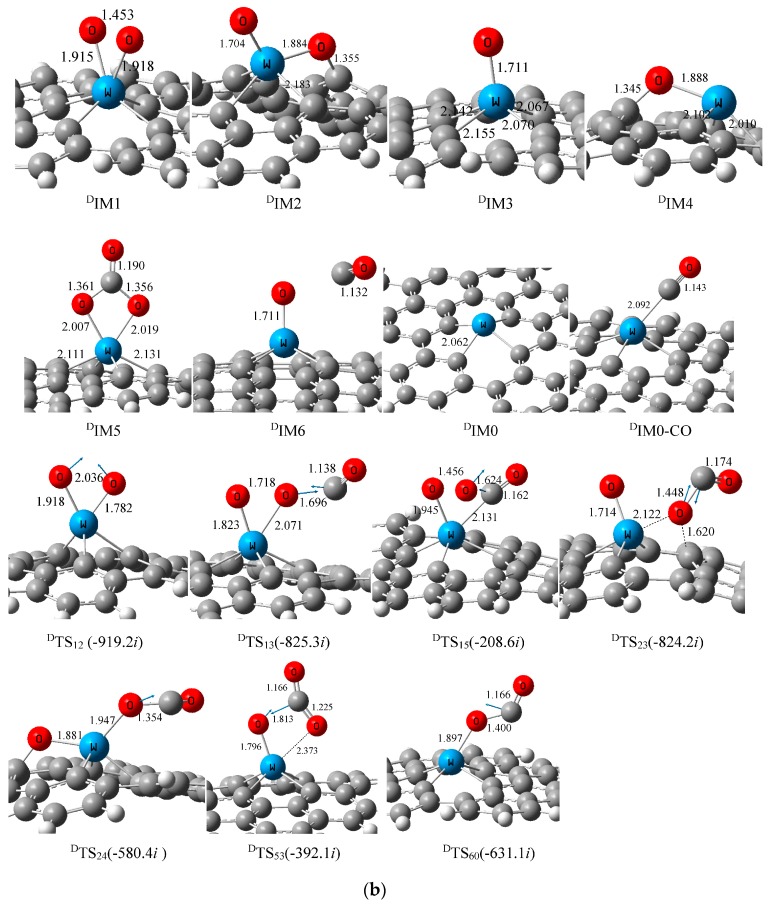
(**a**) Optimized structures over W-SV-graphene (distances in Å). (**b**) Optimized structures over W-DV-graphene (distances in Å).

First, the geometric structures of tungsten atom adsorbed on single and double vacancy (SV and DV) of defective graphene were investigated, as summarized in Figure 1. The stability of various configurations was evaluated by their relative energy and migration barriers. When a tungsten atom is placed above a single or double vacancy in graphene, it moves outward beyond the graphene plane because the tungsten has a radius larger than the carbon atom. The calculation results indicate that tungsten atom combines strongly with defect graphene in these two cases, the stability of W-graphene was confirmed by the high diffusion barriers of 65.74 and 125.17 kcal mol^−1^, respectively. So the tungsten atom could be stabilized both at the single and double carbon defects on the graphene substrate. According to the ^S^IM0 structure in Figure 1a, tungsten forms a tetrahedral configuration with three neighboring carbon atoms, and the average W–C bond length is 1.948 Å, which is in good agreement with the bond length of 1.94 Å obtained by Tang [32]. According to our calculations, there was a strong interaction between the tungsten atom and the surface, as the calculated adsorption energy of embedded atom on the single vacancy of graphene was as high as −188.52 kcal mol^−1^, which is in good accordance with the results of −156.8 and −200.6 kcal mol^−1^ obtained by Zhang [31] and Tang [32], respectively. After comparing the calculated bond length of W–C and adsorption energy of tungsten atom over single carbon defect on the graphene with previous theoretical results, we believe the computational model and theoretical method chosen in this work are reliable for exploring the mechanism of CO oxidation over W-embedded graphene.

**Figure 2:**
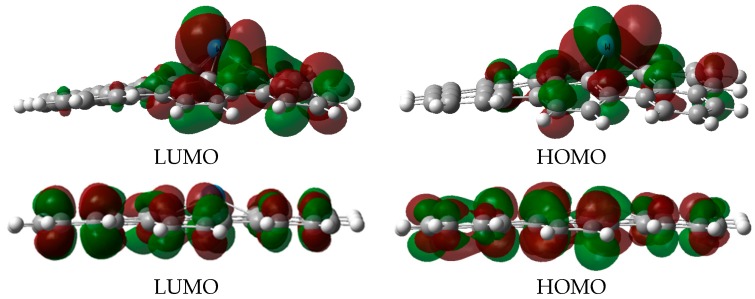
Frontier molecular orbits of W-SV-graphene and W-DV-graphene.

**Figure 3:**
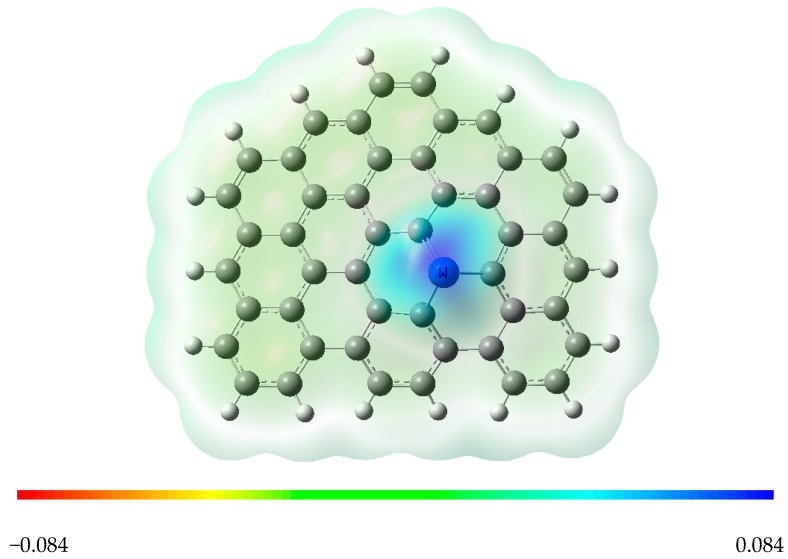
Molecular electrostatic potential (MEP) surface of W-SV-graphene.

Figure 2 shows the frontier molecular orbitals of ^S^IM0, which can help us understand the adsorption properties of tungsten on graphene vacancies. As can be seen from Figure 2, tungsten and adjacent carbon atoms contribute greatly to the HOMO and LUMO of W-SV-graphene. Both the HOMO and LUMO electron clouds of this compound exhibit strong *d* orbital property of the tungsten atom, which will not only provide *d* electrons for the adsorbed molecule, but also allow one electron to occupy this LUMO. The above observations indicate tungsten may be the activated site for the adsorption of electrophilic probe molecules. In addition, the calculated molecular electrostatic potential (MEP) map of this graphene composite molecule shows the electrophilic region near the metal site (see Figure 3). The more positively charged tungsten site is expected to interact with gas molecule strongly. In order to further analyze the interaction between molecules, NBO [36] analysis was carried out by using the optimized geometry and showed that in W-SV-graphene there is −0.778 e electron transfer from tungsten to adjacent carbon due to the different electronegativity between tungsten and carbon atoms. In the case of W-DV-graphene, the tungsten and four adjacent carbon atoms form a pentahedron structure with an average W–C bond length of 2.062 Å (^D^IM0). The calculated adsorption energy of tungsten atom on the double vacancy is −238.1 kcal mol^−1^, indicating much stronger interaction between the inpurity atom and surface in W-DV-graphene. Molecular orbital analysis and NBO results show that this configuration has electronic properties similar to that of the W-SV-graphene.

### 3.2. Adsorption of O_2_ and CO Species over W-Embedded Graphene

**Figure 4:**
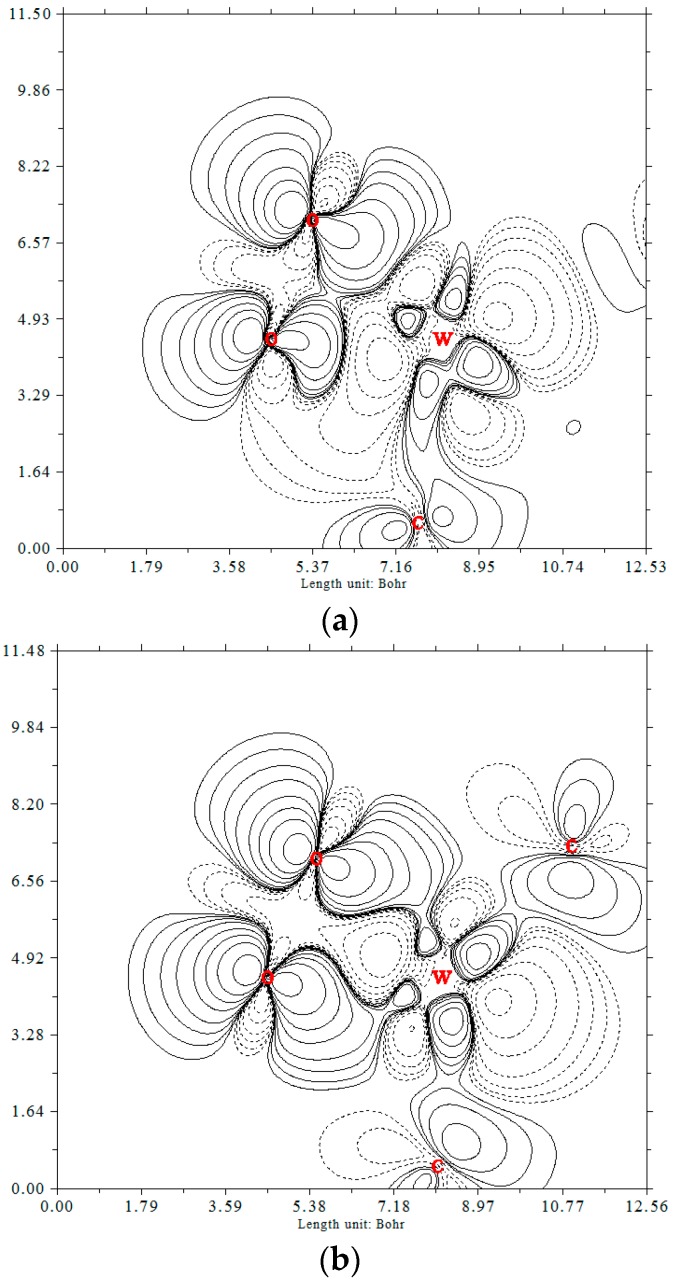
Electron density difference (EDD) between the oxygen and (**a**) W-SV-graphene; (**b**) W-DV-graphene (The electron density accumulation and depletion sites are displayed in solid and dashed lines, respectively).

The adsorption behavior of gas molecules on the surface of catalyst could play a significant role in subsequent catalytic reactions. The most stable adsorption configurations for O_2_ and CO on W-graphene were obtained, as summarized in Figure 1. The adsorbed oxygen molecule prefers to lie and parallel to the plane of W-SV-graphene, and forms two bonds with tungsten atom to form intermediate ^S^IM1 with the adsorption energy of −79.26 kcal mol^−1^. The NBO analysis indicates, the charge transfer from W-embedded graphene to O_2_ is about −0.578 e (see Table 1), occupying the antibond π* orbital of O_2_, resulting in the O–O bond elongation from 1.197 to 1.433 Å. Over W-DV-graphene, the O_2_ is also parallel to the surface, and the O–O bond is enlarged to 1.453 Å. NBO analysis shows that the charge transfer behavior in this case is similar to that of W-SV-graphene. As shown in Table 2, O_2_ molecule extracts about −0.622 e charge from the catalyst surface, so the O_2_ (ads) forms superoxide on both surfaces, and it can become an active center for subsequent reactions. Figure 4 shows the electron density difference (EDD) plots of the adsorbed configurations, which can help us understand the charge transfer behavior between the adsorbed molecules and the surface. The EDD plots were obtained using the MultiWFN electronic structure package [37]. In Figure 4, the full and dotted lines are related to the electron density accumulation and depletion areas respectively. The EDD plot shows in both cases, the charge transfer from the surfaces to oxygen, and the accumulation of electron density at the two W–O bonds. The EDD plots are in good agreement with the NBO analysis.

With respect to CO, the calculated adsorption energies are −25.39 and −18.14 kcal mol^−1^ on the W-SV-graphene and W-DV-graphene surface, respectively, which are much weaker than that of O_2_. Figure 1 gives the most stable CO adsorption configuration. The results indicate that CO could be adsorbed on the surface of the two catalysts with the binding distances of 2.101 and 2.092 Å, respectively. According to NBO analysis results, there is a weak charge transfer between CO and the surface. In both cases, the electrons of CO enter into the *sd* hybrid orbital of W, and the *d* electrons of W feed back into the π* orbital of CO. The net result is that the charges of −0.088 and −0.246 e of CO molecule transfer to the surface of W-SV-graphene and W-DV-graphene respectively, which is different from the adsorption of O_2_, the surface play a role of electron withdrawing here. Compared with free CO (1.132 Å), the bond lengths of the two adsorption configurations have no significant change (1.148 and 1.143 Å, respectively).

It is well known that there are two feasible mechanisms for CO oxidation, named Eley-Rideal (ER) mechanism and Langmuir-Hinshelwood (LH) mechanism. In the ER mechanism, the adsorbed O_2_ molecule is activated first, then the CO in gas phase gets closer to the reactive oxygen species to generate CO_2_ directly. While in the latter, O_2_ and CO co-adsorb on the surface and form CO_2_ directly. As mentioned above, the adsorption of O_2_ on W-embedded graphene surface is much stronger than CO, suggesting that O_2_ molecule will preferentially adsorb on the two surfaces. Therefore, the tungsten site will be mainly covered by the oxygen molecule if a mixture of O_2_ and CO is injected as the reactants, and the formation of O_2_-W-graphene complex (IM1) can be considered as the initial step of CO oxidation.

### 3.3. The CO Oxidation on Tungsten-Embedded Graphene through ER Mechanism

The ER mechanism over the W-SV-graphene is discussed first, that is, the activation of O_2_ and followed by the oxidation of CO as presented in Equations (2) and (3).

O_2_→O_2_ads→O_2_act (2)

O_2_act + CO→Oads + CO_2_ (3)

**Figure 5:**
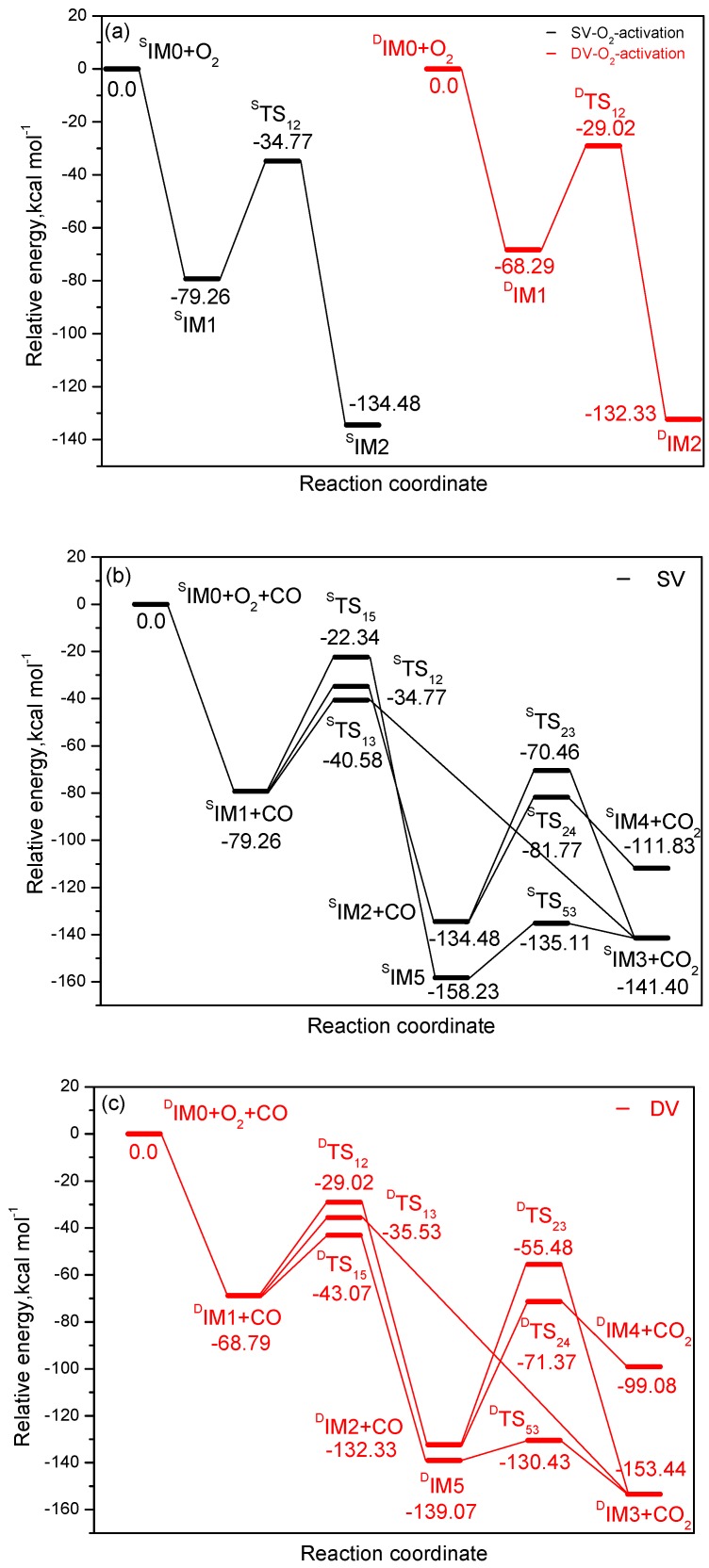

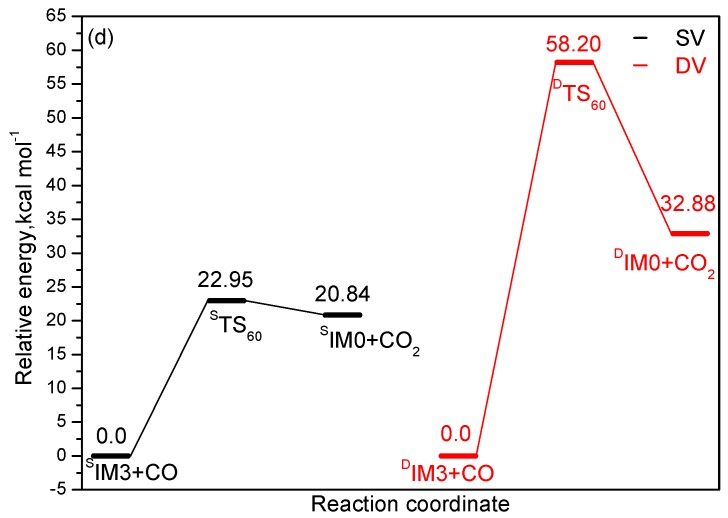
Energy profiles for the reactions of (**a**) O_2_ activation; (**b**) CO oxidation over W-SV-graphene; (**c**) CO oxidation over W-DV-graphene; (**d**) CO oxidation on W-embedded graphene by Oads.

The relative Gibbs free energy Δ*G* of all species is provided in Figure 5. Figure 5a described the energy curve of O_2_ activation. This reaction starts with the formation of O_2_-W-graphene (^S^IM1) complex, which is −79.26 kcal mol^−1^ below ^S^IM0 + O_2_. ^S^IM1 can rearrange to form ^S^IM2 with an energy barrier of 44.49 kcal mol^−1^ (^S^TS_12_). As shown in Figure 1a, the O–O bond in ^S^TS_12_ is almost broken as the bond length is elongated to 2.036 Å. In ^S^IM2, the activated O_2_ molecule forms two adsorbed oxygen atoms on the surface: one is located above the tungsten site while the other co-binds with the tungsten and a neighboring carbon atom, named Otop and Ostep respectively. NBO analysis shows that the negative charges on these two O are −0.330 and −0.248 e, respectively. The O^−^ species is formed, which is beneficial to the adsorption of CO and other probe molecules. The calculation shows that the activation reaction of oxygen over W-DV-graphene follows a similar mechanism with that on the W-SV-graphene, both reactions are greatly exothermic processes and spontaneous in energy. According to NBO analysis, a large number of electrons on the catalyst surface are transferred to oxygen species, the catalyst plays the role of electrons donor in both cases. Starting from ^S^IM2, when CO attacks oxygen atoms at different adsorption sites, there are two possible ways to generate CO_2_ through ER mechanism. When CO attacks Otop in complex ^S^IM2, ^S^IM4 and CO_2_ can be generated after overcoming the activation energy of 52.71 kcal mol^−1^ (^S^TS_24_). Alternatively, if the carbon atom in CO attacks Ostep, it can also produce CO_2_ by overcoming ^S^TS_23_, leaving the Otop adsorbed on the tungsten site. However, the activation energy of this step is quite high (64.02 kcal mol^−1^). Apparently, on the surface of W-SV-graphene, Otop has stronger activity than Ostep toward CO oxidation. 

According to NBO results, the charge on carbon monoxide is increased to 0.100 (^S^TS_24_) and 0.245 e (^S^TS_23_) respectively, whereas the charge on the graphene is decreased from −0.119 e in ^S^IM2 to −0.150 (^S^TS_24_) and −0.330 e (^S^TS_23_) respectively. Therefore, the graphene surface acts as an electron acceptor during the oxidation of CO, which is different from that of O_2_ activation over W-embedded graphene. The desorption of CO_2_ will leave atomic O species on the surface. (see ^S^IM3 and ^S^IM4). The oxygen in ^S^IM3 and ^S^IM4 can extract charge from tungsten easily due to its strong electronegativity. According to the NBO analysis, in ^S^IM3 and ^S^IM4, there are 1.022 and 1.002 e on the W sites, while −0.531 and −0.603 e on the oxygen atoms, indicating the W–O site might be the active centre for adsorbing and oxidating CO, which will be discussed in the later section.

The third feasible ER oxidation pathway was identified by calculation, as described in Figure 5b. Through transition state ^S^TS_15_ with an activation energy of 56.92 kcal mol^−^^1^, the carbon atom in CO inserts into the O–O bond of preadsorbed oxygen molecule to form a peroxo-type species ^S^IM5 (OOCO), which is the most stable species on the PES with the relative energy of −158.23 kcal mol^−1^. Along the reaction coordinate, after passing the transition state ^S^TS_53_, one of the W–O bonds and a C–O bond break simultaneously to produce ^S^IM3 and CO_2_, this reaction requires 23.12 kcal mol^−^^1^ activation energy. From Figure 5b, this CO oxidation channel ^S^IM1 + CO→^S^IM5→^S^IM3 + CO_2_ is less feasible in energy than the two ER oxidation mechanisms discussed above. The NBO analysis shows that during the whole process of CO oxidation, the positive charge change on tungsten atom from ^S^IM1 to ^S^IM3 is not distinct, so the metal centre only acts as a bridge for transmitting electrons. 

Three possible reaction pathways of CO oxidation over W-DV-graphene using ER mechanism were identified also, as shown in Figure 5c. In general, the ER mechanisms on this surface are similar to that on the SV surface. However, unlike the SV surface, the third channel, i.e., when CO inserts into adsorbed oxygen to form OOCO species, followed by removing of CO_2_ is the most feasible as it requiring lowest activation energy (^D^TS_15_, 25.72 kcal mol^−^^1^) on this surface. After comparing the potential energy curves on two different surfaces in Figure 5b,c, the catalytic activity of DV surface is stronger than that of W-SV-graphene.

### 3.4. The CO Oxidation on Tungsten-Embedded Graphene through LH Mechanism

In a previous research about CO oxidation over Sn-embedded graphene, Esrafili et al. [14] located the favorable structure for co-adsorbed O_2_ + CO system. Though in the structure the oxygen chemisorbs on the Sn-graphene while the CO physically adsorbs on the Sn site, their calculated results indicate the LH reaction as the first step is more feasible rather than the ER reaction. Obviously, the adsorption properties of CO do not determine the mechanism of oxidation. In this study, the co-adsorption of O_2_ and CO over two different W-embedded graphene were obtained, with the adsorption energy of −76.62 and −64.71 kcal mol^−^^1^, respectively, which are close to the adsorption of oxygen alone, indicating the oxygen and carbon monoxide molecules may co-adsorb on the tungsten site, so the possibility of CO oxidation using LH mechanism should be considered also.

The LH mechanism of CO oxidation on the surface of W-SV-graphene was studied firstly, and a feasible reaction channel was determined. As shown in Figure 5b, when O_2_ and CO are co-adsorbed on the surface, CO extracts one oxygen atom from the O_2_ molecule which has not been activated completely to produce ^S^IM3 and CO_2_ directly. The energy barrier (^S^TS_13_) of this step is 38.6 kcal mol^−1^. From Figure 5b, it can be seen that the CO oxidation channel ^S^IM0 + O_2_ + CO→^S^IM1 + CO→^S^TS_13_→^S^IM3 + CO_2_ via LH mechanism is spontaneous in energy and exothermic by −141.40 kcal mol^−1^, so this mechanism is the most feasible CO oxidation channel on the surface of W-SV graphene. Similarly, CO has a possible oxidation path on the W-DV-graphene surface using the LH mechanism. After the transition state ^D^TS_13_, CO extracts one oxygen atom from the pre-adsorbed oxygen to generate carbon dioxide and ^D^IM3 directly, but the activation energy of this step is higher than that of ^D^TS_15_ (33.26 kcal mol^−1^ vs. 25.72 kcal mol^−1^). So it is more feasible to adopt ER mechanism to oxidize CO on the surface of W-DV-graphene.

From Figure 5a to Figure 5c, if ER mechanism is used on the W-SV-graphene surface, the adsorbed oxygen must be activated first through ^S^TS_12_ or ^S^TS_15_. As the activation barrier of these steps are high, reaching 44.49 kcal mol^−1^ or 56.92 kcal mol^−^^1^, so the activation of O_2_ is rather difficult. If the LH mechanism is adopted, the pre-adsorbed oxygen will react with CO to produce carbon dioxide directly, the energy barrier of rate-determining step during ^S^IM1 + CO→^S^IM3 + CO_2_ is 38.68 kcal mol^−^^1^, so for the CO oxidation over W-SV-graphene surface, the LH mechanism is more feasible. On the contrary, over the surface of W-DV-graphene, CO inserts and activates pre-adsorbed oxygen molecule to form peroxide species OOCO and then dissociates into carbon dioxide and ^D^IM3, which is the most feasible of all CO oxidation channels, so the ER mechanism is dominant on this surface. The above calculations also show that W-DV-graphene has higher catalytic activity than W-SV-graphene for CO oxidation, regardless of ER or LH mechanism.

### 3.5. The CO Oxidation on Tungsten-Embedded Graphene by Oads

From the above calculations, it can be seen that when CO_2_ is released through either ER or LH mechanism, an O atom is left on the surface of W-embedded graphene. On either surface, the product IM3, that is, the oxygen species on the top of the metal (named Otop), can be obtained through the most feasible oxidation pathway. Now turn to the second CO oxidation pathway involved by Otop, which may lead to catalyst recovery. From Figure 5d, after adsorption of CO over W site, through ^S^TS_60_, the carbon, tungsten and Otop form a three-membered ring transition state, the product (CO_2_ + ^S^IM0) is yielded at last after overcoming activation barrier of 22.95 kcal mol^−^^1^. For an excellent catalyst, its recovery and reuse is of utmost importance. The present calculation shows that the catalyst has good recovery as the surface can be recovered after second CO oxidation. Of course, in the actual production, some advanced catalyst recovery methods such as membranes, will help to recover the catalyst effectively [38]. In addition, the possibility of using the leaving Otop for CO oxidation on the W-DV-graphene surface was also checked. As descripted in Figure 5d, the mechanism on W-DV-graphene is different from that on W-SV-graphene, CO in the gas phase can directly extract the Otop species on the surface and generate products (CO_2_ + ^D^IM0). This reaction requires a high activation energy (58.20 kcal mol^−^^1^), suggesting the remaining Otop species on W-DV-graphene has weaker activity toward CO oxidation.

The above results show that the surface of pure W-DV-graphene has stronger activity for CO oxidation than W-SV-graphene. After a period of reaction, the surface metal sites will be covered by a certain amount of Otop. Although this Otop species remaining on the surface also has a certain oxidation capacity toward CO, the oxidation activity of Otop over W-SV-graphene surface is significantly higher than that on W-DV-graphene surface, so the recovery of the latter surface is more difficult. Therefore, it is important to control the ratio of SV to DV on W-graphene surface in the design of catalyst to achieve the best catalytic activity.

Up to date, compared with the surface of SV-graphene, there are few studies on the CO oxidation over DV-graphene surface. In the present research, the CO oxidation reactions over the two different tungsten-embedded graphene have been explored comprehensively and compared. The results show the catalytic oxidation of carbon monoxide over the two embedded graphene surfaces follows different mechanisms, these two surfaces show different catalytic activities in different reaction stages. Previous experiment has confirmed that both single vacancy (SV) and double vacancy (DV) can exist simultaneously on the surface of graphene [24]. In experiment, the graphene was exposed to the focused electron beam to introduce defects. Some important properties such as the location of vacancies and the ratio of SV to DV in graphene can be adjusted by controlling the total dose of the electron beam. The present theoretical calculations, combined with previous experimental studies, provide some clues to the experimental design of catalysts. In the process of catalyst design, the optimum catalytic performance toward CO oxidation can be obtained by controlling specific reaction conditions to adjust the appropriate ratio of SV to DV.

In general, the oxidation of carbon monoxide on embedded graphene has attracted considerable interests over the last few years. After comparing these related studies, it is found that both noble metals and non-noble metals, and even some non-metallic-embedded graphenes have superior catalytic activity for the oxidation of CO. The present research show that, although not as good as these noble metals, tungsten-embedded graphene also has a high catalytic activity for CO oxidation. Considering the low cost and high activity, we believe tungsten-embedded graphene is also a good catalyst toward CO oxidation, which can be used as a gas sensor candidate for monitoring and removing toxic gases.

## 4. Conclusions

The CO oxidation reactions by O_2_ over W-SV-graphene and W-DV-graphene have been investigated using the DFT approach. The LH mechanism is preferred for CO oxidation on a W-SV-graphene surface with an energy barrier of 38.68 kcal mol^−1^, while over W-DV-graphene, the catalytic process is likely to proceed with the ER mechanism, the calculated energy barrier is only 25.72 kcal mol^−1^. During different reaction processes, the catalyst substrate plays different roles of electron donor or acceptor. For the second CO oxidation, the Otop over W-SV-graphene surface shows significantly higher activity than that on W-DV-graphene surface with the energy barrier of 22.95 kcal mol^−1^ and 58.20 kcal mol^−^^1^, respectively. As the two surfaces exhibit different catalytic activities during different reaction stages, the ratio of SV to DV should be adjusted reasonably in the process of catalyst design to obtain the best catalytic performance.

## Figures and Tables

**Table 1:** Calculated NBO charge of different species over W-SV-graphene.

Species	W	O1	O2	Sum(O_2_)	C	O	Sum(CO)	Sum(Graphene)
^S^IM0	0.778	–	–	–	–	–	–	−0.778
^S^IM0 + CO	0.484	–	–	–	0.512	−0.424	0.088	−0.572
^S^CO		–	–	–	0.517	−0.517	0	–
^S^IM1	1.072	−0.330	−0.248	−0.578	–	–	–	−0.494
^S^IM2	1.256	−0.549	−0.588	−1.137	–	–	–	−0.119
^S^IM3	1.022	−0.531	–	–	–	–	–	−0.491
^S^IM4	1.002	–	−0.603	–	–	–	–	−0.399
^S^IM5	1.108	−0.601	−0.658	−1.259	1.034	−0.580	0.454	−0.303
^S^IM6	0.712	−0.486	–	–	0.700	−0.376	0.324	−0.550
^S^TS_12_	1.213	−0.289	−0.484	−0.773	–	–	–	−0.440
^S^TS_13_	1.273	−0.475	−0.458	−0.933	0.725	−0.457	0.268	−0.608
^S^TS_15_	0.813	−0.294	−0.346	−0.640	0.795	−0.595	0.200	−0.373
^S^TS_23_	1.230	−0.552	−0.593	−1.145	0.713	−0.468	0.245	−0.330
^S^TS_24_	1.268	−0.662	−0.556	−1.218	0.638	−0.538	0.100	−0.150
^S^TS_53_	1.115	−0.619	−0.649	−1.268	1.112	−0.479	0.633	−0.480
^S^TS_60_	0.852	−0.630	–	–	0.709	−0.440	0.269	−0.491

**Table 2:** Calculated NBO charge of different species over W-DV-graphene.

Species	W	O1	O2	Sum(O_2_)	C	O	Sum(CO)	Sum(Graphene)
^D^IM0	0.875	–	–	–	–	–	–	−0.875
^D^IM0 + CO	0.438	–	–	–	0.661	−0.415	0.246	−0.684
^D^CO	–	–	–	–	0.517	−0.517	0	–
^D^IM1	1.290	−0.312	−0.310	−0.622	–	–	–	−0.668
^D^IM2	1.440	−0.530	−0.583	−1.113	–	–	–	−0.327
^D^IM3	1.321	−0.535	–	–	–	–	–	−0.786
^D^IM4	1.020	–	−0.611	–	–	–	–	−0.409
^D^IM5	1.291	−0.665	−0.660	−1.325	1.083	−0.589	0.494	−0.460
^D^IM6	1.298	−0.537	–	–	0.518	−0.507	0.011	−0.772
^D^TS_12_	1.226	−0.474	−0.276	−0.750	–	–	–	−0.476
^D^TS_13_	1.274	−0.473	−0.396	−0.869	0.711	−0.464	0.247	−0.652
^D^TS_15_	0.797	−0.319	−0.306	−0.625	0.753	−0.475	0.278	−0.450
^D^TS_23_	1.302	−0.504	−0.575	−1.079	0.689	−0.537	0.152	−0.375
^D^TS_24_	1.258	−0.647	−0.585	−1.232	0.753	−0.533	0.220	−0.246
^D^TS_53_	1.299	−0.627	−0.659	−1.286	1.114	−0.519	0.595	−0.608
^D^TS_60_	1.077	−0.623	–	–	0.732	−0.502	0.230	−0.684

## Supplementary Materials

The following are available online at http://www.mdpi.com/1996-1944/11/10/1848/s1.
