# Supplementary material for: Tungsten-Embedded Graphene: Theoretical Study on a Potential High-Activity Catalyst toward CO Oxidation
Source: Materials (Basel). 2018 Sep 28;11(10):1848. doi: 10.3390/ma11101848 (PMC6213162; doi:10.3390/ma11101848)
Supplement: Supplementary file 1 [file materials-11-01848-s001.pdf]

Supporting Information

The coordinates of species involved in the reaction

^{S}IM0

C	0.40774100	-5.16673000	0.21461900
C	1.87273300	-5.14933700	0.20763600
C	2.57171400	-3.93726400	0.47470100
C	1.84333000	-2.66600400	0.42986100
C	0.42245000	-2.68809700	0.20587800
C	-0.28944700	-3.92728800	0.16834800
C	3.99614800	-4.04527200	0.63235700
C	2.48874100	-1.40949100	0.46968900
C	3.88117400	-1.30045400	0.82091900
C	4.63195200	-0.12034300	0.47879400
C	3.92181400	1.06810000	0.18227300
C	2.49143100	1.02916600	0.08000200
C	1.78246200	-0.20564900	0.17793500
C	0.38366500	-0.23436900	0.00392400
C	-0.29705400	-1.47963100	0.04769900
C	2.60982900	-6.28111700	-0.15360600
C	3.99886100	-6.27151000	-0.24890000
C	4.73999400	-5.16084200	0.15145100
C	6.21969600	-5.09048400	0.03901400
C	6.92301600	-3.85329900	0.21005800
C	6.26410400	-2.66742100	0.65893500
C	6.05503300	-0.14212600	0.45305200
C	6.82759300	-1.37209600	0.50141500

C	1.77767900	2.22464100	-0.16911200
C	4.61913900	2.29008800	0.00744000
C	6.73933400	1.09962700	0.24584900
C	8.25489800	-1.32364200	0.26460000
C	8.31574800	-3.75976600	-0.05719700
C	6.97111500	-6.20408900	-0.32021900
C	-0.31847000	-6.35109900	0.24874000
C	-1.71110500	-3.92804400	0.07025400
C	-1.72926200	-1.49903800	-0.09449700
C	-0.32799700	0.98897500	-0.21013500
C	2.50659400	3.45265300	-0.30276200
H	1.94486800	4.36503200	-0.48414400
C	3.86407000	3.48499200	-0.22394900
H	4.40101800	4.42241800	-0.34146100
C	6.02220000	2.27568700	0.06612000
C	8.18617800	1.10966400	0.19251900
C	8.95493900	-2.49109600	0.03965600
H	10.02534900	-2.43763100	-0.14530400
C	8.90373400	-0.03139700	0.19506300
C	9.02179300	-4.92599000	-0.42495500
H	10.08803200	-4.86035200	-0.62367100
C	8.35680400	-6.12709300	-0.53663200
C	0.37256500	2.17844600	-0.29095200
C	-1.76714200	0.93178300	-0.34017300
H	-2.30503500	1.86395400	-0.49017500
C	-2.42710400	-0.24295000	-0.27736400
H	-3.50875500	-0.27694000	-0.37542000

C	-2.40159500	-2.69638100	-0.05734700
H	-3.48533800	-2.71084900	-0.14739400
C	-2.40588000	-5.15722800	0.08757000
H	-3.49026200	-5.14613200	0.02263500
C	-1.71909400	-6.34576200	0.19173300
H	-2.25845300	-7.28708400	0.22452100
H	9.98627100	-0.00855400	0.10291500
H	8.68149600	2.07303700	0.10516200
H	6.56545600	3.20829900	-0.06966600
H	-0.16965400	3.10520900	-0.46601900
H	8.89943400	-7.02582000	-0.81182000
H	0.19820600	-7.30017500	0.33981100
H	2.08358100	-7.17735300	-0.46232000
H	4.48879300	-7.15292100	-0.64924400
H	6.48910400	-7.16812800	-0.43897900
W	4.72074100	-2.68357300	1.85637600

^{S}IM0-CO

C	0.44216600	-5.14554600	0.07893600
C	1.90531500	-5.12635800	0.18161500
C	2.57016800	-3.93017100	0.54477500
C	1.84264800	-2.65818100	0.46104800
C	0.42826000	-2.67169000	0.21305500
C	-0.26919800	-3.91265000	0.08887900
C	3.96590600	-4.00283700	0.85698900
C	2.48520500	-1.40316100	0.47089100
C	3.89138400	-1.27300600	0.71831800

C	4.66326100	-0.10081000	0.39703500
C	3.92806900	1.10265500	0.20917300
C	2.49222100	1.05631300	0.17476300
C	1.78141200	-0.18355500	0.25129300
C	0.38127200	-0.20917700	0.10363800
C	-0.29320000	-1.45942700	0.09492600
C	2.67539900	-6.26240600	-0.10537500
C	4.05845800	-6.22193600	-0.06935800
C	4.75269400	-5.10126800	0.41009700
C	6.24836700	-5.06256100	0.29517600
C	6.96406600	-3.82138700	0.18069900
C	6.31622600	-2.59167400	0.45484500
C	6.08776300	-0.11305900	0.24318900
C	6.88221900	-1.32828100	0.20620500
C	1.76770300	2.25574500	-0.01144900
C	4.61359100	2.32274200	0.00392400
C	6.75179400	1.13536700	-0.00101700
C	8.28010500	-1.29197800	-0.14785800
C	8.33881500	-3.76850700	-0.17199900
C	7.00518300	-6.21852300	0.15059000
C	-0.27614700	-6.33242800	-0.01271200
C	-1.68636200	-3.91576100	-0.05570800
C	-1.72356000	-1.47990500	-0.06470900
C	-0.33838600	1.02056000	-0.05164400
C	2.48733100	3.48778900	-0.14444100
H	1.91601000	4.40355200	-0.27039000
C	3.84757500	3.52238500	-0.14877500

H	4.37578100	4.46264000	-0.27982100
C	6.02170800	2.31046100	-0.07379100
C	8.18630200	1.13875400	-0.22696900
C	8.95647600	-2.48736000	-0.30730300
H	10.01506500	-2.45800000	-0.55856600
C	8.90783100	0.00187200	-0.31715500
C	9.04095900	-4.98395200	-0.34145400
H	10.09554600	-4.96466700	-0.60175800
C	8.38104200	-6.17781100	-0.15439200
C	0.35770800	2.21250000	-0.09666300
C	-1.77861800	0.96159200	-0.17341000
H	-2.32297400	1.89682300	-0.27093100
C	-2.42952600	-0.21962400	-0.17678400
H	-3.51094100	-0.25530500	-0.27661300
C	-2.38490700	-2.68232000	-0.11443000
H	-3.46662900	-2.69942100	-0.22605300
C	-2.37004000	-5.14611000	-0.15574900
H	-3.45163400	-5.13868500	-0.25695700
C	-1.67305800	-6.33308900	-0.12403100
H	-2.20226700	-7.27857800	-0.18729200
H	9.97295700	0.03664200	-0.52908800
H	8.66599300	2.10380300	-0.36588300
H	6.54427400	3.24821500	-0.24805500
H	-0.18780000	3.14452700	-0.22676300
H	8.92075600	-7.11399000	-0.25649100
H	0.24185300	-7.28375700	0.02576600
H	2.19388000	-7.16373500	-0.46554900

H	4.60928100	-7.07487900	-0.45327600
H	6.53622800	-7.18940500	0.27106500
W	4.89459000	-2.46397300	1.81632600
C	4.58699100	-2.91486200	3.84497000
O	4.37255900	-3.29180800	4.90795100

^{S}IM1

C	-2.90524200	-3.17822500	-0.17888100
C	-1.44192200	-3.15842900	-0.19334800
C	-0.75519800	-1.95245400	0.11764300
C	-1.46475900	-0.67446300	0.16929500
C	-2.90507800	-0.69642700	0.04720000
C	-3.60405300	-1.93874400	-0.07123600
C	0.65849700	-2.06406700	0.30373100
C	-0.81946400	0.59620000	0.24438300
C	0.59890900	0.76818700	0.43661800
C	1.30894900	1.96277900	0.13834200
C	0.55586000	3.13631800	-0.05024000
C	-0.87783900	3.06572800	-0.01962000
C	-1.56325700	1.81768700	0.10063000
C	-2.96738600	1.77206800	0.02487200
C	-3.63688900	0.51151100	0.02835100
C	-0.67082600	-4.27152200	-0.54579500
C	0.72646600	-4.27669200	-0.54789600
C	1.43575100	-3.17267300	-0.07706300
C	2.91072100	-3.05577300	-0.02637800
C	3.56175500	-1.76137800	0.06966000

C	2.88323100	-0.54888100	0.38520600
C	2.71779100	1.94534800	0.06575000
C	3.47310000	0.69912200	0.11265900
C	-1.62161600	4.25901000	-0.16559100
C	1.22691300	4.36599000	-0.25633500
C	3.37333900	3.18362100	-0.20064300
C	4.87503600	0.74964700	-0.20457900
C	4.96443600	-1.68825200	-0.20903800
C	3.69446900	-4.18143900	-0.21075200
C	-3.62856300	-4.36042100	-0.25447100
C	-5.02941000	-1.94301200	-0.10860900
C	-5.07302500	0.48921900	-0.03745100
C	-3.70658700	2.99244800	-0.07974500
C	-0.92024700	5.50315400	-0.33441500
H	-1.50963600	6.41026700	-0.43564200
C	0.43613400	5.55719200	-0.38787300
H	0.94521400	6.50627800	-0.53041100
C	2.62905100	4.35677100	-0.32497200
C	4.80941800	3.18653900	-0.37806300
C	5.59171900	-0.42597200	-0.29647800
H	6.66277500	-0.38763700	-0.48098200
C	5.51242100	2.03550700	-0.39631900
C	5.70883300	-2.87934700	-0.40283100
H	6.77670200	-2.80078600	-0.58753900
C	5.09130200	-4.10301900	-0.36797200
C	-3.02755300	4.19617500	-0.16530100
C	-5.15022200	2.92758900	-0.10882500

H	-5.70321200	3.86121300	-0.16083900
C	-5.79427400	1.74368200	-0.07917700
H	-6.87940700	1.70074400	-0.10632400
C	-5.73272400	-0.71583900	-0.07696000
H	-6.81928300	-0.73457500	-0.11443400
C	-5.72193200	-3.17181200	-0.19923200
H	-6.80785300	-3.15859000	-0.21783200
C	-5.03117000	-4.35856000	-0.26158400
H	-5.56593300	-5.30111200	-0.31775400
H	6.58485100	2.04589600	-0.57136500
H	5.30350300	4.14117000	-0.53556400
H	3.15282900	5.29306500	-0.50391000
H	-3.59533200	5.11937800	-0.25860000
H	5.66570300	-5.01360200	-0.50217800
H	-3.11058000	-5.31215400	-0.28833800
H	-1.17473900	-5.16889900	-0.88773900
H	1.23884100	-5.15599500	-0.92421200
H	3.22352700	-5.15805400	-0.25245700
W	1.15098300	-0.65435000	1.58088200
O	2.39837400	-0.62986000	3.01859900
O	1.07020300	-0.70318000	3.552507001

^{S}IM2

C	-2.87104900	-3.13845900	-0.34893500
C	-1.41389400	-3.08911900	-0.21722800
C	-0.79538600	-1.91107400	0.26509200
C	-1.52868000	-0.64630700	0.28606500

C	-2.94522900	-0.67990700	0.00101000
C	-3.60696700	-1.92289300	-0.23703300
C	0.57811300	-1.98140700	0.63920200
C	-0.90969700	0.63319100	0.39935700
C	0.47935800	0.77380300	0.71503700
C	1.25735800	1.93692100	0.31355900
C	0.48837000	3.11586700	0.11040300
C	-0.95204300	3.07303800	0.11558300
C	-1.64668700	1.83461400	0.17890600
C	-3.04223100	1.78423200	0.02393200
C	-3.69176900	0.51691400	-0.04989600
C	-0.57950300	-4.15370400	-0.56804500
C	0.80408400	-4.08532800	-0.43995600
C	1.44060500	-3.01502600	0.20624800
C	2.94517500	-2.94959200	0.20481600
C	3.67559700	-1.69914000	0.10430900
C	3.27951100	-0.46742600	0.69192500
C	2.67425600	1.95559600	0.12000000
C	3.52229800	0.75637800	0.05663200
C	-1.69765300	4.26449200	-0.04486500
C	1.13482300	4.35514900	-0.14036100
C	3.26249900	3.18420200	-0.30582400
C	4.71501600	0.77855500	-0.75203400
C	4.87547900	-1.65083600	-0.66685300
C	3.66216100	-4.09809900	-0.08287400
C	-3.55631200	-4.32589400	-0.56009700
C	-5.02196700	-1.94793100	-0.40438800

C	-5.11633400	0.47961100	-0.23099200
C	-3.78357900	2.99758100	-0.09383300
C	-1.00089800	5.51507700	-0.18541600
H	-1.58851200	6.42425300	-0.27691300
C	0.35097500	5.55629500	-0.24824400
H	0.87315800	6.49783700	-0.39296800
C	2.51339200	4.35837500	-0.34268800
C	4.60624100	3.20519600	-0.84438700
C	5.40722900	-0.39144400	-1.01495900
H	6.36713200	-0.34267900	-1.52203600
C	5.25677400	2.06299900	-1.13482400
C	5.52565800	-2.87070600	-0.99661900
H	6.47004200	-2.83290600	-1.53163600
C	4.96856600	-4.06395200	-0.62335300
C	-3.10164200	4.20419800	-0.11071300
C	-5.22152100	2.91954700	-0.21950300
H	-5.78301000	3.84775200	-0.27706700
C	-5.84964000	1.72698000	-0.27954400
H	-6.92948200	1.67376100	-0.38639500
C	-5.74839300	-0.73397600	-0.37464300
H	-6.82791100	-0.76712600	-0.50322400
C	-5.67603000	-3.18207800	-0.62387700
H	-6.75616800	-3.18829600	-0.73888700
C	-4.95373500	-4.34963500	-0.69263900
H	-5.45879400	-5.29695900	-0.85078400
H	6.21811400	2.07785300	-1.64013600
H	5.02871800	4.16982600	-1.11015000

H	3.00586000	5.28743400	-0.62025000
H	-3.66170300	5.13062700	-0.21599200
H	5.48027200	-4.99831700	-0.82899100
H	-3.01258100	-5.26314000	-0.60360400
H	-1.00487900	-5.03628000	-1.03315800
H	1.39119200	-4.88468500	-0.87773000
H	3.18386800	-5.06488000	0.02984500
W	0.99629400	-0.56946700	2.01097900
O	2.87996300	-0.50638600	2.03259000
O	-0.19479900	-0.73208400	3.24494500

^{S}IM3

C	0.44768800	-5.12088400	0.12532900
C	1.91748700	-5.08276900	0.17304500
C	2.60076100	-3.87703700	0.47600100
C	1.81450700	-2.63530100	0.44576200
C	0.40257200	-2.65738200	0.21398500
C	-0.28440000	-3.90112300	0.11574600
C	4.02057100	-3.91765900	0.66966200
C	2.42359500	-1.37840600	0.47099900
C	3.80709400	-1.29350600	0.72329400
C	4.62590000	-0.14816700	0.40369000
C	3.90282500	1.07425900	0.22834200
C	2.46585700	1.05743000	0.16933400
C	1.73433500	-0.16483400	0.23894500
C	0.34020700	-0.19250600	0.08069800
C	-0.32488200	-1.44744000	0.08571600

C	2.67160800	-6.22689400	-0.11760700
C	4.04981300	-6.19731300	-0.11310600
C	4.76717100	-5.05956300	0.29468700
C	6.25835400	-5.09878800	0.19655000
C	6.99252800	-3.87992700	0.13124400
C	6.31083100	-2.68920900	0.41472400
C	6.05499000	-0.17332200	0.27564700
C	6.85482500	-1.39441300	0.25752900
C	1.75209400	2.26063000	-0.03166000
C	4.60458100	2.28691400	0.04966000
C	6.73307100	1.08251100	0.10835000
C	8.27985600	-1.34096900	-0.01170200
C	8.37678800	-3.81254100	-0.15207300
C	7.00194600	-6.26153700	0.04454900
C	-0.26200200	-6.31621400	0.08517700
C	-1.70270800	-3.91463400	-0.01097900
C	-1.75379900	-1.47604100	-0.07153700
C	-0.37085500	1.03836500	-0.09129400
C	2.49215300	3.48074500	-0.15311300
H	1.94028100	4.40636800	-0.29310500
C	3.85381000	3.49423200	-0.12294800
H	4.39715500	4.42774200	-0.23918100
C	6.01569600	2.26343100	0.02744300
C	8.17676100	1.09182800	-0.04103200
C	8.98050000	-2.51757700	-0.19620300
H	10.04829600	-2.45736500	-0.39695500
C	8.90595100	-0.04001400	-0.11420200

C	9.07423100	-5.02741700	-0.32812100
H	10.14033200	-5.01700600	-0.53580800
C	8.38905300	-6.21896400	-0.20871100
C	0.33957100	2.22422300	-0.13702300
C	-1.81071100	0.97057900	-0.22184300
H	-2.36139400	1.90017000	-0.33623200
C	-2.45750200	-0.21486700	-0.20764700
H	-3.53867300	-0.25190000	-0.31038100
C	-2.40843000	-2.68454800	-0.09420100
H	-3.49065100	-2.71080000	-0.19911400
C	-2.37385200	-5.15444300	-0.06578900
H	-3.45664300	-5.16416900	-0.15269800
C	-1.66120700	-6.33147200	-0.00343600
H	-2.18085700	-7.28402300	-0.02720100
H	9.98026500	0.00205200	-0.27065200
H	8.65963800	2.06060600	-0.13395100
H	6.55342500	3.19855400	-0.10856200
H	-0.19570600	3.16043300	-0.27927600
H	8.92468200	-7.15666700	-0.31784700
H	0.26158700	-7.26324400	0.14467100
H	2.17497000	-7.13826000	-0.42848500
H	4.58516800	-7.07831000	-0.45038400
H	6.52808100	-7.23360700	0.12642100
W	4.94534500	-2.37756400	1.81227200
O	4.44594800	-3.29141800	3.20631200

^{S}IM4

C	-2.90182000	-3.09216300	-0.33571800
C	-1.45691000	-3.08331200	-0.10429000
C	-0.81806200	-1.88088700	0.19885800
C	-1.52276700	-0.60269800	0.25006700
C	-2.93860600	-0.62408400	0.01035000
C	-3.61320800	-1.85819500	-0.25096600
C	0.56201300	-1.91911200	0.51418700
C	-0.88288600	0.61750600	0.48623300
C	0.53713300	0.80224100	0.94370200
C	1.27308000	1.93499800	0.28376700
C	0.52025400	3.11779100	0.15927600
C	-0.91346800	3.09117100	0.31470100
C	-1.60586100	1.83887000	0.37008900
C	-3.00954100	1.81646900	0.21494300
C	-3.67813700	0.57818200	0.05382500
C	-0.67104200	-4.27287000	-0.14639600
C	0.68375300	-4.27600400	0.07622700
C	1.37253900	-3.06751600	0.36892800
C	2.85424800	-2.98696500	0.44636400
C	3.54489700	-1.73126800	0.23109500
C	2.88411800	-0.47502300	0.33770900
C	2.62268500	1.91397100	-0.12359500
C	3.41868700	0.71260100	-0.15528300
C	-1.64690700	4.28697800	0.20786900
C	1.15600700	4.33665800	-0.20584100
C	3.23665600	3.12813100	-0.56441200
C	4.77780500	0.70099200	-0.57696100

C	4.94983100	-1.70119300	-0.08417100
C	3.63906600	-4.13515000	0.57293500
C	-3.60805400	-4.25861300	-0.60921900
C	-5.02653400	-1.85430100	-0.43568600
C	-5.10763700	0.56137700	-0.11952400
C	-3.74530500	3.04795600	0.18374000
C	-0.95232700	5.52765100	-0.00145300
H	-1.53414100	6.44433600	-0.03663900
C	0.38509200	5.54643800	-0.22856900
H	0.89702900	6.47944000	-0.44822300
C	2.51214800	4.31349600	-0.55180900
C	4.61539000	3.09464100	-1.00430700
C	5.52473100	-0.47320000	-0.48776200
H	6.57657200	-0.45718400	-0.75995900
C	5.34537500	1.95605100	-1.01835800
C	5.66480000	-2.92085300	-0.01259900
H	6.72531100	-2.92455200	-0.24706000
C	5.02476200	-4.08594600	0.35499900
C	-3.06101600	4.24402700	0.21084600
C	-5.18741500	2.99050500	0.07574700
H	-5.73736000	3.92736200	0.09310000
C	-5.83113800	1.81481300	-0.06731300
H	-6.91248900	1.78038500	-0.16627300
C	-5.74324300	-0.63198800	-0.35021900
H	-6.82202500	-0.65101900	-0.48768000
C	-5.69740300	-3.06513900	-0.70448800
H	-6.77488300	-3.04743100	-0.84193000

C	-4.99595400	-4.24678300	-0.79344900
H	-5.51472800	-5.17634200	-1.00400000
H	6.37899000	1.96749600	-1.35333200
H	5.05838200	4.03156000	-1.33229200
H	3.00002700	5.24024500	-0.84696500
H	-3.61466200	5.17895100	0.16087400
H	5.59741300	-5.00514600	0.43634500
H	-3.09002000	-5.20744200	-0.67975500
H	-1.14260600	-5.21337600	-0.40373200
H	1.23675300	-5.20046500	-0.05504600
H	3.17185600	-5.08963400	0.78721200
W	1.57483400	-0.80496500	1.73995000
O	0.69220800	0.78174700	2.34309500

^{S}IM5

C	3.28063100	1.65926600	4.48272300
C	4.74366500	1.67909200	4.47021700
C	5.42889200	2.89006300	4.76828200
C	4.71939200	4.16460500	4.81555500
C	3.27822400	4.14106400	4.70077600
C	2.58126500	2.89820000	4.58722900
C	6.84203600	2.78203900	4.95931600
C	5.36406700	5.43386100	4.88894400
C	6.78362600	5.60513100	5.08002600
C	7.49086400	6.80057800	4.77635300
C	6.73548000	7.97307600	4.59389000
C	5.30277000	7.90390500	4.63169100

C	4.61963200	6.65619100	4.75365300
C	3.21570100	6.60940600	4.68322700
C	2.54689400	5.34849400	4.68630600
C	5.51623500	0.56396700	4.13222800
C	6.91383800	0.56120300	4.13458200
C	7.62279400	1.66921400	4.59350200
C	9.09409100	1.78697700	4.65402300
C	9.73848700	3.08361800	4.73930500
C	9.04629800	4.29474600	5.02492800
C	8.89768700	6.78365400	4.70605300
C	9.64836900	5.53645600	4.75866600
C	4.55855600	9.09719100	4.49094500
C	7.40595500	9.20313700	4.38983200
C	9.55414100	8.02216900	4.44422700
C	11.05518000	5.58866500	4.46542000
C	11.14589900	3.15396100	4.48558100
C	9.87980900	0.65807200	4.49539600
C	2.55753400	0.47689700	4.41033500
C	1.15614800	2.89300100	4.55338900
C	1.11076500	5.32594000	4.62538800
C	2.47501600	7.82912600	4.58290400
C	5.25925800	10.34192000	4.32203700
H	4.66982300	11.24938800	4.22573500
C	6.61533800	10.39520200	4.26407800
H	7.12464500	11.34435500	4.12336200
C	8.80793700	9.19418900	4.32055300
C	10.99129000	8.02542600	4.27868700

C	11.77545100	4.41452800	4.39589100
H	12.84960100	4.45181300	4.23087900
C	11.69385500	6.87398600	4.27475600
C	11.89372700	1.96178000	4.31777500
H	12.96454000	2.03922200	4.15067600
C	11.27771500	0.73684700	4.35746800
C	3.15276300	9.03366500	4.49690700
C	1.03123500	7.76373300	4.55861700
H	0.47780100	8.69717300	4.51055600
C	0.38801700	6.57948800	4.58802600
H	-0.69708500	6.53568600	4.56490100
C	0.45271700	4.11990600	4.58627200
H	-0.63394800	4.10034000	4.55229500
C	0.46356900	1.66413000	4.46601000
H	-0.62233500	1.67679800	4.45002000
C	1.15486000	0.47771000	4.40466300
H	0.62089500	-0.46532300	4.35136800
H	12.76849200	6.88386500	4.11413300
H	11.48695100	8.97949000	4.12351600
H	9.33104800	10.13170200	4.14593900
H	2.58402300	9.95656100	4.40787300
H	11.85600200	-0.17412400	4.24485500
H	3.07525300	-0.47498400	4.37836500
H	5.01517300	-0.33875800	3.80085100
H	7.42911400	-0.32248000	3.77299700
H	9.41045300	-0.31966800	4.46324400
W	7.29789900	4.18740700	6.23971800

O	8.75838700	4.19166200	7.57647800
O	6.66429400	4.10048200	8.15540300
C	7.91755100	4.11810600	8.66154100
O	8.25184000	4.07916500	9.80334000

^{S}IM6

C	-2.84429800	-3.11756800	-0.52612700
C	-1.38324100	-3.07088000	-0.47892800
C	-0.72408200	-1.90499900	-0.02436900
C	-1.46566600	-0.61767900	0.03927400
C	-2.89574100	-0.65950000	-0.14710000
C	-3.57191200	-1.90715600	-0.35372400
C	0.66854700	-2.01480800	0.26766700
C	-0.85302600	0.65797100	0.14813500
C	0.55054700	0.92915700	0.47775200
C	1.22645500	2.11416000	0.07576300
C	0.45266300	3.26502200	-0.13074100
C	-0.97581000	3.14989600	-0.07718400
C	-1.61963800	1.86915700	0.01307200
C	-3.02415300	1.79866400	-0.08438600
C	-3.66235600	0.53126900	-0.13368500
C	-0.59947700	-4.16963900	-0.87946300
C	0.78110200	-4.15069300	-0.82104500
C	1.44054000	-3.07555100	-0.20323300
C	2.91672400	-2.95176000	-0.19928800
C	3.52202800	-1.63824800	-0.22962300
C	2.81654500	-0.44345200	0.08169700

C	2.61656500	2.07784600	-0.14968800
C	3.37128700	0.82183000	-0.20047000
C	-1.74462200	4.32220300	-0.22324900
C	1.09275800	4.49509600	-0.40277000
C	3.24300500	3.31985700	-0.48140400
C	4.75063200	0.89458400	-0.60388000
C	4.90207000	-1.54005500	-0.58368000
C	3.71886300	-4.06352600	-0.35535300
C	-3.53823100	-4.30945500	-0.70243000
C	-4.99529800	-1.94206600	-0.42510600
C	-5.09898800	0.47755500	-0.22374300
C	-3.79521200	3.00840900	-0.15991400
C	-1.07703800	5.58147600	-0.41308300
H	-1.69150000	6.47285300	-0.50516700
C	0.27575300	5.66800300	-0.51833600
H	0.75627500	6.62614400	-0.69527900
C	2.48996500	4.49077600	-0.55381200
C	4.66014400	3.33165900	-0.77107400
C	5.48485800	-0.26632500	-0.74199400
H	6.53958400	-0.20251300	-0.99990400
C	5.36489000	2.18509600	-0.83732600
C	5.67627700	-2.72008800	-0.75101500
H	6.73139600	-2.61913800	-0.99047500
C	5.10514100	-3.95497700	-0.60343500
C	-3.15236900	4.22734300	-0.22050700
C	-5.23699900	2.91162300	-0.19514300
H	-5.81009200	3.83432200	-0.21540200

C	-5.85216600	1.71364800	-0.21660800
H	-6.93566200	1.64404900	-0.25383400
C	-5.73004400	-0.73502900	-0.33456800
H	-6.81505000	-0.77642600	-0.39321700
C	-5.65768200	-3.17420600	-0.61118600
H	-6.74303200	-3.18230700	-0.65352500
C	-4.93783000	-4.33952900	-0.74038500
H	-5.44915100	-5.28755000	-0.87308800
H	6.42254500	2.20030500	-1.08662900
H	5.13472600	4.28916600	-0.96659400
H	2.99844100	5.42653200	-0.77500800
H	-3.74267600	5.13743700	-0.30139600
H	5.70151700	-4.85508000	-0.71100100
H	-2.99782300	-5.24484000	-0.78792600
H	-1.08479400	-5.03158300	-1.32279400
H	1.34333000	-4.96507800	-1.26691700
H	3.27285200	-5.05223800	-0.30537300
W	1.29419200	-0.46431500	1.53988600
C	-0.09179300	-1.72180500	3.01639500
O	2.50445500	-0.13548600	2.74059700
O	-0.72341700	-2.38002700	3.67414200

$^{S}TS_{12}$

C	-2.88118900	-3.17231000	-0.20058600
C	-1.42091500	-3.15346900	-0.21943200
C	-0.73535400	-1.97186500	0.15605300
C	-1.44188300	-0.68079500	0.24492100

C	-2.87598600	-0.69914200	0.10987400
C	-3.57682400	-1.93990700	-0.03718200
C	0.66620800	-2.10057700	0.37259400
C	-0.79390200	0.60708800	0.28082600
C	0.61160200	0.78717400	0.46194400
C	1.31669300	1.96737100	0.14933900
C	0.56398700	3.13785600	-0.07065000
C	-0.86503500	3.06638700	-0.03636100
C	-1.54476400	1.82130500	0.10863400
C	-2.94944400	1.77361800	0.05907400
C	-3.61219600	0.51197300	0.08867300
C	-0.64708000	-4.25681800	-0.61438900
C	0.74532000	-4.25916700	-0.60134800
C	1.44608400	-3.17910800	-0.05064900
C	2.92228700	-3.05878500	0.00554900
C	3.56888900	-1.75820000	0.08136000
C	2.88751700	-0.54734900	0.39221200
C	2.73142300	1.94439400	0.06779400
C	3.48204800	0.69673900	0.10983600
C	-1.61377500	4.26009000	-0.19382700
C	1.23476200	4.36463600	-0.29518500
C	3.38299400	3.17731300	-0.21717900
C	4.88235700	0.74779200	-0.21641700
C	4.96940100	-1.68899100	-0.21145000
C	3.70425000	-4.18428500	-0.17061400
C	-3.60683600	-4.35203100	-0.31497000
C	-5.00198700	-1.94528100	-0.06313400

C	-5.04707200	0.48804000	0.03719000
C	-3.69259500	2.99017800	-0.05234900
C	-0.91319600	5.50119300	-0.38695700
H	-1.50264500	6.40676400	-0.49934100
C	0.44343200	5.55351200	-0.44581900
H	0.95187700	6.50044700	-0.60419300
C	2.63512300	4.35144600	-0.35793600
C	4.81695200	3.18278300	-0.40219700
C	5.59818700	-0.42972900	-0.31122500
H	6.66725800	-0.39320600	-0.50741100
C	5.52020800	2.03084200	-0.41477500
C	5.71591700	-2.88325200	-0.39517300
H	6.78394400	-2.80477800	-0.57965700
C	5.10240700	-4.10609900	-0.33950000
C	-3.01657600	4.19588800	-0.17286300
C	-5.13468500	2.92379900	-0.05256500
H	-5.69113700	3.85524600	-0.10361400
C	-5.77449300	1.73743400	-0.00005200
H	-6.85965100	1.68998000	-0.00817700
C	-5.70493400	-0.72004600	-0.00016300
H	-6.79202600	-0.73934800	-0.02436700
C	-5.69784700	-3.16880700	-0.18520100
H	-6.78384500	-3.15398300	-0.19012900
C	-5.00828800	-4.35315100	-0.30074800
H	-5.54336400	-5.29332300	-0.38417300
H	6.59252200	2.04087500	-0.59110400
H	5.30935800	4.13675200	-0.56847400

H	3.16104800	5.28409300	-0.55034900
H	-3.58828600	5.11570700	-0.27311900
H	5.67768800	-5.01776800	-0.46252300
H	-3.08707600	-5.30009900	-0.39427900
H	-1.14966700	-5.13459700	-1.00582300
H	1.26900300	-5.11025500	-1.02473400
H	3.23253800	-5.16149300	-0.19553000
W	1.11580900	-0.65605200	1.66624400
O	2.27372000	-0.60913300	3.01999400
O	0.27413800	-0.72221500	3.38831500

$^{S}TS_{13}$

C	-2.84147600	-3.17846000	-0.11319000
C	-1.37622800	-3.13576300	-0.07374600
C	-0.70818600	-1.94105000	0.28324200
C	-1.46590700	-0.67828800	0.27982700
C	-2.88559200	-0.70912900	0.08366800
C	-3.56928200	-1.95778800	-0.04493500
C	0.69965400	-1.99533500	0.51474700
C	-0.84444200	0.58615300	0.30523700
C	0.56192200	0.72276600	0.52221300
C	1.31014200	1.91591600	0.24072600
C	0.55552200	3.11218300	0.08270800
C	-0.87824600	3.04857000	0.07149100
C	-1.57047400	1.79863100	0.13531700
C	-2.97254200	1.75478000	0.02474200
C	-3.62822500	0.49575500	0.01375300

C	-0.59937800	-4.25526900	-0.41479900
C	0.78135200	-4.20738800	-0.42509600
C	1.47502300	-3.08478400	0.06160600
C	2.96845700	-3.03021600	-0.06415400
C	3.66639000	-1.77799100	-0.13801300
C	3.01944400	-0.57211700	0.21572300
C	2.73403400	1.91588000	0.06654200
C	3.54395800	0.71281700	-0.02443200
C	-1.62153600	4.24153600	-0.07452700
C	1.22192500	4.34293500	-0.10414800
C	3.37825700	3.18192500	-0.15747500
C	4.93066800	0.77918100	-0.42196300
C	5.02497000	-1.68946900	-0.53833100
C	3.73180500	-4.17061700	-0.28043900
C	-3.54576900	-4.37413400	-0.20540800
C	-4.99016900	-1.97829200	-0.13829800
C	-5.06263500	0.45755800	-0.09987900
C	-3.71313900	2.97821000	-0.08681900
C	-0.92031000	5.48384700	-0.19755200
H	-1.50390400	6.39506100	-0.29616200
C	0.44044200	5.53476900	-0.22367800
H	0.95481900	6.48370200	-0.34619100
C	2.63145100	4.34593300	-0.19736200
C	4.80663700	3.21130400	-0.41388600
C	5.62270100	-0.39546400	-0.64089500
H	6.67347300	-0.33961900	-0.91717300
C	5.53604700	2.08676000	-0.56383400

C	5.73201100	-2.88702600	-0.78632600
H	6.77445400	-2.84229900	-1.08869300
C	5.09571000	-4.09828600	-0.62510700
C	-3.03405100	4.17936900	-0.12791200
C	-5.15551300	2.89993500	-0.16698700
H	-5.71608800	3.82826000	-0.23204000
C	-5.78930100	1.70944700	-0.16874400
H	-6.87263700	1.65961800	-0.23494100
C	-5.70751600	-0.75357800	-0.14845100
H	-6.79203300	-0.78492500	-0.22326100
C	-5.65972400	-3.21574800	-0.24005200
H	-6.74427400	-3.22240900	-0.30067700
C	-4.94550300	-4.39311000	-0.26163700
H	-5.46350000	-5.34455700	-0.32624400
H	6.59416000	2.14060200	-0.80425300
H	5.27168700	4.18608400	-0.53159700
H	3.14100800	5.29306800	-0.35910400
H	-3.59485600	5.10616100	-0.22587900
H	5.64407200	-5.02093500	-0.78580500
H	-3.01425200	-5.31877600	-0.21185400
H	-1.08559000	-5.15684400	-0.76919000
H	1.32507300	-5.05260600	-0.83422100
H	3.27739000	-5.15165700	-0.19160600
W	1.62869700	-0.44218600	1.60472700
O	1.25359700	-1.47726900	3.27399000
O	2.15623500	0.02042900	3.34464300
C	0.49179700	-2.93175300	3.35969300

O	0.35918100	-3.65885000	4.22680900

$^{S}TS_{15}$

C	-2.95607000	-3.19575100	-0.21977500
C	-1.49269300	-3.17507300	-0.22689000
C	-0.79585100	-1.97084800	0.07472000
C	-1.52086700	-0.69390800	0.12102400
C	-2.95353500	-0.71607800	-0.04408700
C	-3.65585800	-1.95696500	-0.15697500
C	0.62598200	-2.08959000	0.23282800
C	-0.88154200	0.56863900	0.22789600
C	0.52343700	0.72010000	0.52395700
C	1.24632900	1.91581000	0.24850700
C	0.50746000	3.09848300	0.04653700
C	-0.92524000	3.03852800	0.01426500
C	-1.61547600	1.78920700	0.07114700
C	-3.01612800	1.74965400	-0.06061200
C	-3.68504100	0.49371400	-0.08687300
C	-0.74018300	-4.29713300	-0.59223200
C	0.65197100	-4.31188200	-0.62410100
C	1.37749800	-3.20630400	-0.18045900
C	2.84979000	-3.10562400	-0.17766900
C	3.49907600	-1.82256100	-0.03970500
C	2.82072600	-0.63362000	0.35961000
C	2.65517300	1.88351200	0.16237400
C	3.40428300	0.63195100	0.14483900
C	-1.65827100	4.23561100	-0.14368400

C	1.18773300	4.32730500	-0.12314300
C	3.32196200	3.12662300	-0.06277000
C	4.80793400	0.69442900	-0.17147300
C	4.89544300	-1.73467100	-0.33246300
C	3.62525500	-4.22393200	-0.43974100
C	-3.67854900	-4.38117400	-0.27030200
C	-5.07967500	-1.96020800	-0.22332700
C	-5.11998500	0.47293800	-0.19112800
C	-3.74897600	2.97504100	-0.17392400
C	-0.94787500	5.47909500	-0.25835000
H	-1.52842200	6.39115600	-0.36566800
C	0.41062400	5.52515200	-0.25937400
H	0.93056300	6.47298800	-0.36702900
C	2.59098500	4.30801300	-0.16040800
C	4.75939300	3.13430900	-0.22617200
C	5.52417000	-0.47035000	-0.34493600
H	6.59321100	-0.41913900	-0.53669400
C	5.45637600	1.98372300	-0.29094100
C	5.63370500	-2.91225800	-0.60611600
H	6.69843700	-2.82363100	-0.80396500
C	5.01605300	-4.13762400	-0.62697800
C	-3.06532900	4.17662100	-0.20932500
C	-5.19072100	2.91227700	-0.25853000
H	-5.74047400	3.84694000	-0.32474500
C	-5.83689600	1.72917000	-0.25786800
H	-6.92051600	1.68807000	-0.32260200
C	-5.78104800	-0.73014700	-0.23312400

H	-6.86643400	-0.74761800	-0.29651500
C	-5.77111600	-3.18966100	-0.29389000
H	-6.85642300	-3.17702900	-0.33493700
C	-5.07986500	-4.37876600	-0.30361600
H	-5.61451900	-5.32236400	-0.33941400
H	6.53034600	1.99376400	-0.45551400
H	5.25675700	4.09395700	-0.33506500
H	3.12619500	5.24415800	-0.30325200
H	-3.62579000	5.10344900	-0.31051100
H	5.58694800	-5.03920300	-0.82239000
H	-3.16140800	-5.33383800	-0.26293300
H	-1.25988200	-5.18811000	-0.92637800
H	1.15099900	-5.19852500	-0.99983200
H	3.15359400	-5.19765800	-0.51745200
W	1.12965200	-0.75098900	1.58544500
O	3.44904700	-0.18679500	3.48817600
O	2.41740100	0.78190600	2.94504100
C	2.38545300	-0.87175700	3.13487400
O	1.46776000	-1.43959700	3.76543100

$^{S}TS_{23}$

C	3.25649500	1.69446000	4.40026600
C	4.71344000	1.72675700	4.53871100
C	5.34664600	2.90057100	5.01288100
C	4.62372600	4.17494100	5.01389300
C	3.21344400	4.15640800	4.71243700
C	2.53505900	2.91856000	4.48785300

C	6.72022200	2.81224200	5.38167500
C	5.25722800	5.44443600	5.11905600
C	6.64845800	5.57645000	5.44805000
C	7.42326900	6.72685200	5.04147300
C	6.68044300	7.91548400	4.80153100
C	5.24434800	7.88627600	4.78717400
C	4.54006700	6.65220300	4.86824500
C	3.14432600	6.61871600	4.69565500
C	2.48040000	5.36236400	4.63647400
C	5.52131000	0.63323300	4.20422500
C	6.90132800	0.67066100	4.33928600
C	7.54584100	1.74983800	4.96136000
C	9.03924300	1.79872100	4.99577900
C	9.74043600	3.05104100	4.91773200
C	9.11524900	4.27891300	5.25724400
C	8.84609400	6.72625000	4.87100200
C	9.63762100	5.51556600	4.84187800
C	4.51708400	9.08378700	4.59411400
C	7.35488000	9.13977500	4.55779200
C	9.48561300	7.95810700	4.51783400
C	10.97758800	5.54017900	4.31636800
C	11.08094100	3.08933200	4.43649700
C	9.78848400	0.63937300	4.84659200
C	2.55640500	0.51094600	4.20603800
C	1.12113500	2.90836800	4.31226800
C	1.05550700	5.33886700	4.44428800
C	2.41846700	7.84181600	4.54700500

C	5.23297500	10.31778800	4.44040600
H	4.66047000	11.23296700	4.31753600
C	6.58975900	10.34344300	4.40821500
H	7.12353200	11.27775300	4.25922400
C	8.75000500	9.13231400	4.43232200
C	10.89009300	7.96278000	4.16690400
C	11.67787100	4.35372000	4.19133600
H	12.72066900	4.38464700	3.88306600
C	11.58841200	6.81786600	4.02953200
C	11.78067600	1.87387700	4.24823200
H	12.81016500	1.90169900	3.90217100
C	11.15555500	0.67707600	4.50952400
C	3.11114100	9.03902000	4.51343300
C	0.98043000	7.77918700	4.41119700
H	0.43173800	8.71346700	4.33239200
C	0.33798300	6.59412900	4.36838900
H	-0.74165900	6.55209900	4.25458200
C	0.40931100	4.13285400	4.31683000
H	-0.66961700	4.11088400	4.18122100
C	0.45255800	1.68073100	4.11062800
H	-0.62693600	1.68607600	3.98977200
C	1.16095100	0.50233200	4.06723000
H	0.64452800	-0.44133900	3.92438500
H	12.62416200	6.83155000	3.70173800
H	11.35120900	8.92248500	3.95135200
H	9.26310700	10.06229800	4.19852800
H	2.56274100	9.96942000	4.38495900

H	11.69887900	-0.25603200	4.40056500
H	3.08701600	-0.43420900	4.18373300
H	5.07321700	-0.24446600	3.75213100
H	7.48396400	-0.14992400	3.93384400
H	9.30711500	-0.32747600	4.95103000
W	7.28403900	4.23913900	6.71856500
O	9.15629400	4.37369300	7.21107000
O	6.03631900	3.99177600	7.89866300
C	9.71348400	4.52379100	8.62767300
O	10.82151800	4.61717700	8.92386500

$^{S}TS_{24}$

C	-2.95059400	-3.17006300	-0.43135900
C	-1.50431300	-3.16391600	-0.21527400
C	-0.86290700	-2.00806300	0.24969600
C	-1.56168300	-0.69684200	0.25133500
C	-2.97275400	-0.71140900	-0.04662400
C	-3.65642200	-1.93886100	-0.32638800
C	0.50734100	-2.12708700	0.61817900
C	-0.92740900	0.53962700	0.42919100
C	0.46240000	0.74928500	0.97396600
C	1.22089700	1.91657600	0.46280600
C	0.47312900	3.08990100	0.29180700
C	-0.96733200	3.02598300	0.30679600
C	-1.65037200	1.76263000	0.29284000
C	-3.04804500	1.73914100	0.09917700
C	-3.70977000	0.49601700	-0.05580800

C	-0.71531100	-4.31399100	-0.47615200
C	0.65244100	-4.33041000	-0.32607600
C	1.31854200	-3.21396700	0.22371100
C	2.80616700	-3.10502600	0.19093800
C	3.47456900	-1.82001400	0.10160500
C	2.82204100	-0.60248700	0.42845200
C	2.59745100	1.88905200	0.18109500
C	3.36703200	0.64577500	0.08260100
C	-1.69981800	4.21858800	0.17773300
C	1.13083600	4.32089700	0.03441200
C	3.24026100	3.11778400	-0.16551700
C	4.69813700	0.68347300	-0.43643300
C	4.82541700	-1.76015700	-0.35874800
C	3.58328700	-4.24258600	0.04341000
C	-3.65618000	-4.33819200	-0.69643800
C	-5.06697500	-1.93368200	-0.52976900
C	-5.13615100	0.48244200	-0.26156500
C	-3.78586100	2.96960000	0.03773200
C	-1.00264800	5.47308600	0.06127200
H	-1.59463900	6.38192500	-0.00101600
C	0.34871700	5.52342000	-0.03379700
H	0.86196900	6.47039400	-0.17516300
C	2.51371100	4.30618700	-0.16851800
C	4.63140400	3.11278500	-0.55943100
C	5.39908500	-0.50438200	-0.61808200
H	6.43366500	-0.46085600	-0.95035000
C	5.31523900	1.96101700	-0.71412600

C	5.55957400	-2.96806200	-0.50427800
H	6.59919800	-2.91589300	-0.81548900
C	4.95985800	-4.17691200	-0.25925300
C	-3.10978100	4.16852600	0.09358400
C	-5.22275500	2.91369100	-0.11554800
H	-5.77152200	3.85113700	-0.12862400
C	-5.86069600	1.73565700	-0.25508800
H	-6.93879500	1.69665100	-0.38283000
C	-5.77874000	-0.70965100	-0.47261900
H	-6.85551500	-0.72380200	-0.62443100
C	-5.74116500	-3.14312400	-0.79843500
H	-6.81745900	-3.12032600	-0.94334100
C	-5.04486700	-4.32748000	-0.87543900
H	-5.56590300	-5.25829800	-1.07403800
H	6.34783500	1.96723300	-1.05138500
H	5.10123700	4.06908200	-0.77165600
H	3.02768400	5.23917700	-0.38896200
H	-3.66608600	5.10043900	0.02306700
H	5.52743300	-5.09726900	-0.34584000
H	-3.14078700	-5.28986000	-0.74512100
H	-1.18764700	-5.19177500	-0.90065500
H	1.20972800	-5.18952000	-0.68386100
H	3.12598400	-5.22074300	0.15112000
W	1.43537200	-0.85814200	1.85651500
O	2.82487500	-1.27062900	3.08183300
O	0.41016300	0.64646000	2.38859900
C	4.23262700	-1.43447200	3.06737300

O	4.84614300	-1.86000200	3.97574100

$^{S}TS_{53}$

C	3.28483500	1.71346000	4.58793000
C	4.75242600	1.76317600	4.62863000
C	5.42464000	2.96834500	4.95164100
C	4.64035100	4.21265200	4.94786100
C	3.22454000	4.17848300	4.71507200
C	2.54718600	2.92958800	4.60223300
C	6.84314700	2.93453600	5.14808300
C	5.23939200	5.47769800	5.00338400
C	6.62847800	5.59102600	5.27520200
C	7.42266500	6.74661800	4.89507300
C	6.68220400	7.94775700	4.70712300
C	5.24352300	7.91604700	4.68251500
C	4.53167400	6.68386400	4.76920800
C	3.13701500	6.64227000	4.61320700
C	2.48364600	5.38077700	4.60900800
C	5.51758500	0.63209400	4.31552900
C	6.89647400	0.67050900	4.32531000
C	7.59880400	1.80624900	4.76105900
C	9.09013400	1.80021500	4.68528600
C	9.80301900	3.03842000	4.63516300
C	9.11715800	4.22320900	4.94641800
C	8.84279900	6.73003400	4.74749500
C	9.64990900	5.50862700	4.72576900
C	4.51587900	9.10897300	4.48121600

C	7.36508600	9.16822700	4.49294800
C	9.50195400	7.98198700	4.51367500
C	11.05492700	5.57509200	4.38914400
C	11.17858000	3.11347500	4.30155000
C	9.84354500	0.64912800	4.51462000
C	2.58439900	0.51389200	4.53403300
C	1.12795500	2.90415700	4.48496200
C	1.05412500	5.34124300	4.45908100
C	2.40982700	7.86412800	4.44817700
C	5.23842600	10.33988900	4.33323400
H	4.67057200	11.25645400	4.19845700
C	6.59761000	10.36876700	4.32886300
H	7.12931300	11.30606300	4.19145900
C	8.76912400	9.15699200	4.42756100
C	10.93698600	8.00474100	4.31005600
C	11.77023900	4.40415100	4.21482300
H	12.83120200	4.47141300	3.98308300
C	11.66868500	6.87539600	4.23235000
C	11.88867900	1.90554700	4.10704100
H	12.94761000	1.93535800	3.86674800
C	11.22818100	0.70571000	4.24289700
C	3.10454100	9.05893400	4.39532200
C	0.96997100	7.78509800	4.32893500
H	0.41123000	8.71096300	4.22448600
C	0.33457400	6.59415500	4.34007500
H	-0.74678400	6.54565500	4.24528700
C	0.41086900	4.12720400	4.42405900

H	-0.67168400	4.09264200	4.32471000
C	0.46430900	1.66061500	4.41553700
H	-0.61896600	1.64598300	4.33622100
C	1.18461700	0.48803100	4.45424800
H	0.67204900	-0.46794100	4.41929400
H	12.73585200	6.91797900	4.03299900
H	11.40686200	8.97512500	4.17692800
H	9.29487000	10.09338700	4.25670000
H	2.55674400	9.98831500	4.25666100
H	11.77271600	-0.22509800	4.11988300
H	3.11697200	-0.42909100	4.57596600
H	5.02799300	-0.27651800	3.98561300
H	7.43906600	-0.19980100	3.97219100
H	9.37659500	-0.32731200	4.58410200
W	7.69677100	4.43952700	6.34852800
O	9.17460800	4.64568900	8.11785600
O	7.01270000	3.48379600	7.70862100
C	8.52814300	3.99402800	8.91833000
O	8.29896100	3.51555100	9.94795700

$^{S}TS_{60}$

C	-2.84240700	-3.10896900	-0.07022100
C	-1.37559700	-3.06630600	-0.05616500
C	-0.69218300	-1.88465400	0.32149500
C	-1.45829200	-0.61972200	0.34899100
C	-2.88036900	-0.64616800	0.13141800
C	-3.56897500	-1.88914400	-0.00135600

C	0.72838000	-1.95991700	0.53036800
C	-0.85465600	0.65258000	0.41829600
C	0.54140400	0.76795300	0.66316300
C	1.30973800	1.92955700	0.25958500
C	0.57412800	3.12773100	0.08236400
C	-0.86367000	3.09341800	0.08908500
C	-1.57067100	1.85838100	0.19004700
C	-2.96808000	1.82049500	0.05917300
C	-3.62243000	0.55929900	0.05489800
C	-0.62293800	-4.18785700	-0.43592900
C	0.75652900	-4.16639700	-0.44092700
C	1.46767700	-3.06402700	0.05970300
C	2.95341300	-3.05860600	-0.03341600
C	3.64603300	-1.80416700	-0.09970000
C	2.95285300	-0.62105400	0.20099800
C	2.72350700	1.90464000	0.09329600
C	3.51290000	0.66120100	0.03683800
C	-1.59249400	4.28777100	-0.09449200
C	1.25812000	4.34870900	-0.13795400
C	3.38861100	3.14723200	-0.13983800
C	4.91604200	0.72571700	-0.29661000
C	5.02694600	-1.73064400	-0.42344800
C	3.70907300	-4.20338400	-0.19931000
C	-3.54766600	-4.30427300	-0.14523400
C	-4.99038700	-1.90815600	-0.09158900
C	-5.05493500	0.52666800	-0.06925400
C	-3.69722100	3.04375700	-0.08603500

C	-0.87096800	5.52034800	-0.25454900
H	-1.44226200	6.43668700	-0.37570200
C	0.48709300	5.55114900	-0.28449800
H	1.01500900	6.48958700	-0.42876300
C	2.65837200	4.33073700	-0.21557700
C	4.82258500	3.15960700	-0.34822400
C	5.62467600	-0.44766900	-0.49562400
H	6.68613700	-0.38306600	-0.72594400
C	5.54203600	2.02255700	-0.43468400
C	5.74393400	-2.93921400	-0.61346000
H	6.80401900	-2.90072200	-0.84771300
C	5.09525600	-4.14184500	-0.47759500
C	-3.00378800	4.23946900	-0.14982600
C	-5.13877000	2.97010600	-0.17579800
H	-5.69691000	3.89820500	-0.26172100
C	-5.77582200	1.78055200	-0.16010900
H	-6.85888200	1.73321800	-0.23277000
C	-5.70438100	-0.68349900	-0.11143700
H	-6.78871000	-0.71206900	-0.19081200
C	-5.66206000	-3.14634600	-0.18243600
H	-6.74679000	-3.15224200	-0.23963400
C	-4.94883800	-4.32328400	-0.19465500
H	-5.46693500	-5.27540400	-0.24694400
H	6.60967400	2.05609800	-0.63354500
H	5.30324600	4.12579600	-0.47341200
H	3.18914600	5.26463100	-0.38518800
H	-3.55513500	5.16879600	-0.27418100

H	5.64554200	-5.06944100	-0.59809500
H	-3.01728900	-5.24950200	-0.14281400
H	-1.12835400	-5.07178500	-0.80702000
H	1.29166900	-5.01706500	-0.84984600
H	3.24315700	-5.17999300	-0.11801100
W	1.60920200	-0.45138300	1.71443700
C	-0.05681100	-0.90149400	2.97041900
O	1.67798500	-1.00002900	3.48837300
O	-0.90200400	-1.15436100	3.70423700

^{D}IM0

C	0.51397200	-5.57028200	0.31159600
C	1.98252800	-5.55677100	0.28801800
C	2.65977300	-4.33584500	0.40162500
C	1.93094100	-3.07704700	0.42815400
C	0.51451800	-3.08671800	0.35168200
C	-0.18819100	-4.32634500	0.32183600
C	4.07324400	-4.23037600	0.42044700
C	2.67205200	-1.89556200	0.44706000
C	4.92766300	-0.51358700	0.39382000
C	4.14219700	0.65118900	0.27855500
C	2.70151200	0.60141000	0.25352700
C	1.99598100	-0.64409500	0.31930700
C	0.57242200	-0.63921000	0.25259400
C	-0.16355100	-1.85713600	0.28185700
C	2.78370700	-6.69578000	0.09736900
C	4.16104900	-6.60401100	0.05093200

C	4.85321700	-5.38294500	0.23359800
C	6.33420500	-5.34035800	0.20770700
C	7.02726400	-4.08246200	0.24182400
C	6.34792000	-2.84667900	0.36136700
C	6.32817200	-0.41121400	0.39553400
C	7.07897800	-1.65118100	0.38617800
C	1.97466900	1.80786300	0.13717200
C	4.81172300	1.91246400	0.19953100
C	6.99936500	0.83681400	0.33820800
C	8.50319200	-1.62030300	0.32947600
C	8.46186400	-4.05873200	0.18212300
C	7.09116100	-6.49757300	0.14322600
C	-0.22714800	-6.74477200	0.30388600
C	-1.60987000	-4.30283500	0.28296100
C	-1.60103300	-1.85301300	0.22624700
C	-0.14093000	0.59970500	0.15114300
C	2.67565200	3.05789600	0.05980800
H	2.08927600	3.96856300	-0.02568400
C	4.02997600	3.10944700	0.08947700
H	4.55001200	4.06164200	0.02853900
C	6.21695500	1.97985300	0.24037400
C	8.44992100	0.83006200	0.33677400
C	9.16321700	-2.82900900	0.22972100
H	10.24996200	-2.85129100	0.18310100
C	9.15666900	-0.32462900	0.33404900
C	9.17387800	-5.28098800	0.09375000
H	10.25895500	-5.25334100	0.04480900

C	8.49872000	-6.47309900	0.08307600
C	0.56476500	1.78309000	0.09831900
C	-1.58849200	0.57782400	0.10659800
H	-2.10547700	1.53115500	0.03879600
C	-2.28146600	-0.57851600	0.14617100
H	-3.36734900	-0.57863100	0.11095900
C	-2.28099600	-3.04967800	0.24198600
H	-3.36824800	-3.04620700	0.20632100
C	-2.31499100	-5.52592700	0.27243700
H	-3.40097200	-5.50910000	0.24911500
C	-1.63040200	-6.72147200	0.28987500
H	-2.17640700	-7.65946100	0.28755400
H	10.24287200	-0.30139300	0.30804500
H	8.96547400	1.78651000	0.31265000
H	6.69343200	2.95648200	0.18610200
H	0.02659800	2.72494500	0.01806200
H	9.04228400	-7.41085500	0.02993300
H	0.27668000	-7.70519600	0.31569300
H	2.32661700	-7.66871700	-0.04969000
H	4.71245300	-7.51565900	-0.14630800
H	6.60933300	-7.46761400	0.14974100
W	4.54122000	-2.35601900	1.20020300

^{D}IM0-CO

C	0.50194300	-5.56350200	0.22436100
C	1.97133600	-5.54818600	0.26781700
C	2.63502600	-4.33188600	0.46074100

C	1.90004600	-3.07564500	0.47830800
C	0.49183600	-3.08397500	0.35139000
C	-0.20545600	-4.32292000	0.25794600
C	4.03983600	-4.21816000	0.58042700
C	2.64246400	-1.90278100	0.54882900
C	4.95349500	-0.48974300	0.43036800
C	4.14216100	0.65817600	0.33038900
C	2.69479100	0.60452700	0.35820600
C	1.98254800	-0.64131000	0.42694100
C	0.55794200	-0.63238900	0.34018400
C	-0.18065500	-1.84932600	0.31227400
C	2.78901800	-6.67584400	0.06690800
C	4.16605000	-6.56855400	0.08833100
C	4.83523000	-5.35325700	0.37714500
C	6.31832700	-5.30169900	0.40817100
C	7.01831400	-4.04502000	0.35302100
C	6.35152500	-2.79966300	0.45358800
C	6.34107600	-0.37362100	0.31374300
C	7.08669700	-1.61977700	0.32270300
C	1.96454900	1.80940400	0.25229900
C	4.79892800	1.92366400	0.17127300
C	7.00136300	0.86269800	0.15163000
C	8.50269500	-1.59726300	0.17664400
C	8.44889300	-4.03702400	0.22672500
C	7.06971300	-6.46353800	0.44038400
C	-0.23698500	-6.73649400	0.14252700
C	-1.62438000	-4.29764600	0.17807900

C	-1.61643200	-1.84367400	0.22317300
C	-0.15508300	0.60777900	0.25828700
C	2.65666700	3.06418000	0.13898500
H	2.06205600	3.97093700	0.07230300
C	4.00823200	3.11916600	0.09035500
H	4.52194700	4.07091300	-0.01584300
C	6.19937200	2.00009800	0.09200500
C	8.44914800	0.85345600	0.04750200
C	9.15758300	-2.81356800	0.14176300
H	10.24093800	-2.84717200	0.04773600
C	9.15308600	-0.30355100	0.05873800
C	9.15297300	-5.26876000	0.21854500
H	10.23526000	-5.25223600	0.12317300
C	8.47679500	-6.45233600	0.34979700
C	0.55358500	1.78958200	0.22910900
C	-1.60225500	0.58883000	0.19459500
H	-2.11736900	1.54455700	0.15127400
C	-2.29669400	-0.56740300	0.18033400
H	-3.38177500	-0.56529500	0.12637000
C	-2.29355800	-3.04180800	0.17141000
H	-3.37963300	-3.03644800	0.10715500
C	-2.32694000	-5.51930300	0.09384700
H	-3.41167700	-5.50319600	0.03679400
C	-1.63952900	-6.71361700	0.08197000
H	-2.18268200	-7.65128300	0.02151500
H	10.23560400	-0.28475900	-0.03600200
H	8.96381300	1.80522000	-0.05462800

H	6.65987600	2.97903700	-0.02621700
H	0.01883500	2.73410800	0.15935000
H	9.01682100	-7.39331700	0.37393400
H	0.26847000	-7.69626400	0.13310300
H	2.34793000	-7.64257500	-0.15267400
H	4.74166800	-7.45722100	-0.14469200
H	6.57861900	-7.42402500	0.54444600
W	4.52436300	-2.31081800	1.36290200
C	5.30618200	-3.46526500	2.92287000
O	5.74935100	-4.12742700	3.74233000

^{D}IM1

C	0.52544800	-5.55398700	0.11613000
C	1.98681300	-5.53988400	0.27996900
C	2.63148500	-4.33136000	0.53323200
C	1.89039300	-3.07480700	0.55582500
C	0.49710800	-3.08126200	0.33255700
C	-0.18693900	-4.31624500	0.14251400
C	4.03605700	-4.23139100	0.70282300
C	2.60676800	-1.90242500	0.74977000
C	4.97416200	-0.51643800	0.54690300
C	4.14599500	0.62272000	0.47825600
C	2.69499000	0.58507200	0.56984200
C	1.95902300	-0.64871600	0.61913800
C	0.54424200	-0.63297400	0.45417900
C	-0.18284500	-1.84783000	0.32720300
C	2.81985000	-6.67581500	0.16100300

C	4.18906200	-6.58110500	0.27757200
C	4.85570500	-5.35564500	0.56053900
C	6.34553200	-5.30888300	0.58443300
C	7.05810900	-4.06068900	0.44499300
C	6.41578400	-2.81836700	0.54218300
C	6.34883900	-0.41809600	0.26688700
C	7.11553000	-1.65118800	0.25063800
C	1.97124800	1.79432200	0.46896200
C	4.79452600	1.88338800	0.23315300
C	6.97551800	0.80573500	-0.02689200
C	8.49181700	-1.63756000	-0.07510000
C	8.47184200	-4.06997600	0.18345400
C	7.09033400	-6.46897700	0.62851900
C	-0.20155100	-6.72364300	-0.06630200
C	-1.59831000	-4.28879300	-0.02369200
C	-1.61015000	-1.83976800	0.15706100
C	-0.16148400	0.61087900	0.39123400
C	2.66739400	3.04776000	0.34304200
H	2.07770800	3.95946100	0.31224800
C	4.01177300	3.08947900	0.21246600
H	4.52901800	4.03484900	0.07385300
C	6.17167600	1.94949300	-0.00554300
C	8.39338500	0.79527900	-0.33236900
C	9.15372100	-2.86288400	-0.07101000
H	10.22313800	-2.90135300	-0.26815700
C	9.10881300	-0.35651400	-0.35942800
C	9.17030600	-5.31097500	0.20563400

H	10.24252600	-5.31366700	0.02917100
C	8.49806700	-6.47246000	0.46155300
C	0.55858900	1.78648900	0.42960700
C	-1.60400800	0.59406200	0.26022800
H	-2.12180500	1.54902400	0.24080100
C	-2.29125800	-0.56256300	0.14998800
H	-3.37232100	-0.55827100	0.04206300
C	-2.27458600	-3.03549400	-0.00535200
H	-3.35480500	-3.03123600	-0.13497200
C	-2.28792800	-5.50679300	-0.20460300
H	-3.36722600	-5.49103300	-0.32737100
C	-1.59621100	-6.69915100	-0.22284300
H	-2.13056400	-7.63382800	-0.35870700
H	10.16805900	-0.33582700	-0.60097200
H	8.87643000	1.74336000	-0.55279500
H	6.61852700	2.92336100	-0.19585500
H	0.03499500	2.73799000	0.37330800
H	9.03057000	-7.41661500	0.50810400
H	0.30483900	-7.68276400	-0.08149000
H	2.39038300	-7.64618000	-0.06551000
H	4.76888800	-7.48001100	0.10487100
H	6.60389300	-7.42431200	0.78923000
W	4.55237400	-2.34286300	1.47013800
O	5.37664400	-2.47400400	3.19740700
O	3.98054100	-2.07962800	3.27833200

^{D}IM2

C	0.59478100	-5.51993100	0.32345900
C	2.02771400	-5.49718800	0.66040200
C	2.65981700	-4.30237000	1.06133700
C	1.86033400	-3.06549500	1.03044300
C	0.55840400	-3.05356200	0.44269900
C	-0.09159000	-4.28481000	0.14890000
C	4.07993000	-4.27306100	1.25812200
C	2.32487500	-1.84508300	1.49378100
C	5.01999900	-0.54940200	0.92138900
C	4.15463500	0.58603600	0.91752900
C	2.67920700	0.58796400	1.02048400
C	1.85733600	-0.59615300	1.03136800
C	0.54148500	-0.59865100	0.52045300
C	-0.12311800	-1.83019700	0.27152500
C	2.82931500	-6.63684800	0.47218700
C	4.20137800	-6.58180900	0.57445600
C	4.86547500	-5.39048700	0.93234200
C	6.34303400	-5.32422000	0.73635400
C	6.98148600	-4.09246600	0.33818000
C	6.33648100	-2.84873800	0.43963400
C	6.28379400	-0.43830000	0.32394600
C	6.99291600	-1.68120300	0.08060300
C	1.97583400	1.79712800	0.79218600
C	4.77762200	1.84386300	0.57925800
C	6.87275000	0.78062400	-0.06224200
C	8.30633000	-1.68130800	-0.45746500
C	8.33679700	-4.10848800	-0.13559200

C	7.11355200	-6.46790100	0.77032300
C	-0.10945600	-6.69934900	0.11899400
C	-1.44937800	-4.26704900	-0.27826900
C	-1.47996400	-1.82472800	-0.19929400
C	-0.12246800	0.64412100	0.28900000
C	2.67729000	3.04842800	0.71850100
H	2.09681300	3.96498700	0.66927200
C	4.02115500	3.06619600	0.60852500
H	4.55756800	4.00110300	0.47214800
C	6.11034300	1.92338900	0.14998600
C	8.20679100	0.75788200	-0.63229400
C	8.95328200	-2.90660900	-0.55191500
H	9.97629100	-2.95009200	-0.92019900
C	8.88546100	-0.40057500	-0.81699000
C	9.05349000	-5.33642000	-0.12932000
H	10.08177600	-5.34630900	-0.48034700
C	8.46586500	-6.47513200	0.35147700
C	0.58673700	1.80447400	0.50879500
C	-1.49847400	0.61963600	-0.15945300
H	-2.00319400	1.56912800	-0.31393500
C	-2.13834500	-0.54709800	-0.38795400
H	-3.16945600	-0.55427200	-0.73019300
C	-2.10933600	-3.02109400	-0.45522300
H	-3.14048100	-3.02362300	-0.80098800
C	-2.11210200	-5.49449800	-0.49657300
H	-3.15020700	-5.48338200	-0.81619200
C	-1.45382400	-6.68508300	-0.28486800

H	-1.97277900	-7.62677200	-0.43187900
H	9.88625800	-0.38746800	-1.24005300
H	8.65957000	1.70563000	-0.91107100
H	6.52235000	2.90376300	-0.07942700
H	0.09996000	2.76563300	0.36100900
H	9.02685100	-7.40281900	0.39703300
H	0.36944800	-7.65788100	0.28381200
H	2.37689900	-7.56198000	0.13381900
H	4.77617500	-7.45416800	0.28389900
H	6.67919400	-7.39892200	1.12036000
W	4.93553100	-2.46474000	1.96572000
O	6.01668200	-2.61768000	3.27444300
O	3.24371800	-1.82243000	2.48924600

^{D}IM3

C	0.52109100	-5.56481800	0.05914800
C	1.98399900	-5.55742000	0.20077500
C	2.63516600	-4.35006500	0.45504300
C	1.90750800	-3.08946300	0.47401600
C	0.50784200	-3.08954000	0.28198100
C	-0.18435200	-4.32344800	0.10462700
C	4.02852900	-4.24165500	0.63593300
C	2.64463800	-1.92865100	0.64160800
C	4.93333700	-0.42805600	0.50670300
C	4.13679300	0.70441500	0.39573400
C	2.69345500	0.61132100	0.48162300
C	1.99587100	-0.65452000	0.53626100

C	0.57185300	-0.64233400	0.40267900
C	-0.16529700	-1.85506600	0.28988000
C	2.81052700	-6.69229200	0.07068800
C	4.18149900	-6.59245100	0.18014300
C	4.84420000	-5.37078300	0.47802800
C	6.34064100	-5.34612500	0.49868800
C	7.06909100	-4.10924100	0.36764000
C	6.41851400	-2.87941200	0.46101400
C	6.31457900	-0.40454600	0.25475800
C	7.07656400	-1.66521900	0.23891100
C	1.96131000	1.81158900	0.37364400
C	4.79143500	1.94915900	0.13907900
C	6.96418400	0.81832800	-0.03398500
C	8.46985300	-1.64641700	-0.04653400
C	8.48330800	-4.09657800	0.14584000
C	7.09048600	-6.50289000	0.54074000
C	-0.21282400	-6.73098600	-0.11397200
C	-1.59838000	-4.29328400	-0.03354300
C	-1.59781500	-1.84677700	0.15125200
C	-0.14620600	0.59945100	0.34928600
C	2.64899700	3.07000900	0.22127000
H	2.04366100	3.97113500	0.17558600
C	3.99467000	3.14277000	0.08801800
H	4.48920000	4.09770500	-0.06649800
C	6.18115000	1.97706700	-0.05692300
C	8.38914400	0.79131700	-0.30287500
C	9.14261500	-2.86458200	-0.06151800

H	10.21598400	-2.87701000	-0.24001900
C	9.09652700	-0.36431700	-0.30709700
C	9.18474400	-5.33408500	0.16059700
H	10.26014600	-5.33743800	0.00655100
C	8.50110500	-6.49692200	0.38666100
C	0.55075800	1.78742800	0.35811700
C	-1.59179900	0.58120500	0.25364200
H	-2.10696100	1.53777000	0.24572900
C	-2.28231100	-0.57284100	0.16052200
H	-3.36559800	-0.57021200	0.07831200
C	-2.26943300	-3.03893900	0.00234400
H	-3.35222200	-3.02974500	-0.10311200
C	-2.29662600	-5.50775500	-0.20612500
H	-3.37799300	-5.48633600	-0.30806700
C	-1.61020100	-6.70213500	-0.24353100
H	-2.15021400	-7.63452500	-0.37332000
H	10.16101800	-0.35133100	-0.52475600
H	8.88537500	1.73369200	-0.51871400
H	6.65859000	2.93543900	-0.25104300
H	0.00901200	2.72865900	0.30172000
H	9.03330100	-7.44176800	0.42781000
H	0.29151400	-7.69089000	-0.14214700
H	2.38076400	-7.66144800	-0.16031500
H	4.76381600	-7.48782000	-0.00550800
H	6.60822500	-7.46182500	0.69258700
W	4.63597800	-2.29699300	1.33827000
O	4.56512600	-2.35398400	3.04711100

^{D}IM4

C	0.54462100	-5.52495000	0.35237300
C	2.00369500	-5.48520800	0.55976700
C	2.65182200	-4.27616000	0.90112100
C	1.83571900	-3.06962500	0.94252800
C	0.49429600	-3.06319600	0.46100800
C	-0.16836200	-4.29796100	0.22588300
C	4.08425700	-4.19723400	0.98132900
C	2.34769300	-1.85430200	1.36043700
C	4.96884200	-0.58313200	0.62667100
C	4.12651500	0.57098300	0.69060200
C	2.66415100	0.57132000	0.82225700
C	1.84648800	-0.61196200	0.91259900
C	0.49792900	-0.61368300	0.48810800
C	-0.19407300	-1.84319400	0.31489300
C	2.79848500	-6.61038600	0.29977700
C	4.17536800	-6.53567400	0.32441000
C	4.84918700	-5.34240900	0.64374700
C	6.33134800	-5.32729000	0.48556300
C	7.02317700	-4.11025000	0.15673300
C	6.34432000	-2.87621200	0.11906900
C	6.30736200	-0.44775700	0.21335800
C	7.05108100	-1.67680400	0.01357200
C	1.95876600	1.78378300	0.61088400
C	4.77913700	1.83198000	0.45745400
C	6.94823000	0.79760600	0.02200500

C	8.45177800	-1.65374200	-0.25775500
C	8.44019900	-4.10238400	-0.07198900
C	7.09039000	-6.47822000	0.59356900
C	-0.16438600	-6.71137300	0.21469600
C	-1.55412100	-4.28882700	-0.09947400
C	-1.57954000	-1.84467500	-0.06139100
C	-0.16911400	0.62829800	0.26214700
C	2.66887700	3.02652900	0.52191500
H	2.09235800	3.94489900	0.45534500
C	4.01845300	3.04940000	0.46231200
H	4.55481700	3.98819100	0.35622300
C	6.15780900	1.92469600	0.17658000
C	8.35920000	0.79423100	-0.31888700
C	9.10602200	-2.87299500	-0.31952600
H	10.17660400	-2.89890600	-0.51268200
C	9.07063300	-0.35401700	-0.43914100
C	9.14471300	-5.32988900	0.00218700
H	10.21522100	-5.33850900	-0.18284700
C	8.48076100	-6.48160100	0.34400600
C	0.55805900	1.79168100	0.39858600
C	-1.57378400	0.60096900	-0.08970800
H	-2.08128000	1.55021100	-0.23771200
C	-2.23875200	-0.56536300	-0.23744600
H	-3.29146400	-0.56957300	-0.50679900
C	-2.22788600	-3.04484200	-0.24702100
H	-3.28090100	-3.05244400	-0.51884000
C	-2.22038700	-5.52421800	-0.25435300

H	-3.27965000	-5.52604100	-0.49560000
C	-1.53680800	-6.70777400	-0.08238100
H	-2.05902200	-7.65422100	-0.18095400
H	10.12834500	-0.31749000	-0.68530900
H	8.84729500	1.75276200	-0.47419300
H	6.59022200	2.91386700	0.04421600
H	0.06624700	2.75119700	0.25576600
H	9.02746400	-7.41466700	0.43601100
H	0.33461400	-7.66494500	0.34726100
H	2.33627900	-7.53901200	-0.01561900
H	4.74436600	-7.40435000	0.01061900
H	6.61947200	-7.40774000	0.89567100
W	5.03952500	-2.42316400	1.57981700
O	3.36724300	-1.84441000	2.23777200

^{D}IM5

C	0.52646800	-5.55438600	0.10405800
C	1.98602400	-5.54110100	0.26961900
C	2.63226300	-4.32920600	0.49473300
C	1.89871200	-3.07300300	0.49471200
C	0.50306900	-3.08036000	0.29710700
C	-0.18343400	-4.31622300	0.12360200
C	4.03444800	-4.22477600	0.67604400
C	2.62607500	-1.90166800	0.66884200
C	4.95663300	-0.51499300	0.52753200
C	4.13568900	0.62905200	0.47367100
C	2.69004200	0.58880700	0.55475400

C	1.96608200	-0.64869800	0.57487800
C	0.54933400	-0.63386200	0.42782200
C	-0.17858500	-1.84755400	0.29972400
C	2.81544400	-6.68466200	0.18722400
C	4.18186400	-6.58820000	0.31049200
C	4.84943800	-5.35504000	0.56443500
C	6.33297600	-5.30650600	0.58811700
C	7.03198500	-4.05820900	0.42103400
C	6.37567300	-2.82002200	0.48133600
C	6.33115500	-0.41793500	0.24276500
C	7.08632800	-1.65325300	0.20880800
C	1.96420100	1.80019200	0.47756800
C	4.78855700	1.89243900	0.25272000
C	6.96290100	0.80744500	-0.02388800
C	8.46773100	-1.63686900	-0.08986800
C	8.44977500	-4.06487900	0.18296100
C	7.08309800	-6.46267400	0.66426300
C	-0.20271600	-6.72500900	-0.06801900
C	-1.59606100	-4.28957300	-0.03110900
C	-1.60665900	-1.83996000	0.14605800
C	-0.16035400	0.60813300	0.38662100
C	2.66139600	3.05632800	0.37910200
H	2.07211900	3.96858900	0.36646800
C	4.00584500	3.09991700	0.25284400
H	4.52432800	4.04732100	0.13499600
C	6.16404700	1.95726900	0.01872800
C	8.38076400	0.79778100	-0.32255000

C	9.13293300	-2.86067900	-0.07276000
H	10.20565700	-2.89705500	-0.25083400
C	9.09183200	-0.35690800	-0.35658900
C	9.15580600	-5.30041600	0.23437800
H	10.22988100	-5.29767000	0.07003400
C	8.49051700	-6.46249100	0.50663900
C	0.55458300	1.78842700	0.43836200
C	-1.60289900	0.59124900	0.26514700
H	-2.12144500	1.54584800	0.25837900
C	-2.29024400	-0.56522000	0.15092700
H	-3.37209100	-0.56173600	0.05222700
C	-2.27195600	-3.03702600	-0.01039900
H	-3.35344100	-3.03313200	-0.12903700
C	-2.28822200	-5.50758700	-0.20081100
H	-3.36838300	-5.49145800	-0.31530200
C	-1.59773700	-6.70094900	-0.21787300
H	-2.13342100	-7.63586800	-0.34561200
H	10.15341100	-0.33847500	-0.58730900
H	8.86837900	1.74691000	-0.52707700
H	6.61754600	2.93184300	-0.15036900
H	0.02574800	2.73770800	0.39897800
H	9.02919700	-7.40137700	0.57818900
H	0.30278300	-7.68458500	-0.08017900
H	2.38436900	-7.66007900	-0.01201000
H	4.76339200	-7.49113900	0.16786700
H	6.59995700	-7.41549400	0.84810500
W	4.53971400	-2.33360800	1.44798000

O	5.67749200	-2.61625000	3.09187800
O	3.64058800	-1.95369000	3.20170100
C	4.71536500	-2.25946100	3.97874100
O	4.78857100	-2.22124500	5.16546700

DIM6

C	0.52111300	-5.54245900	0.10109400
C	1.98369900	-5.53554900	0.25042300
C	2.64585800	-4.33054300	0.51491800
C	1.90100300	-3.04820200	0.52019300
C	0.49554200	-3.06321400	0.30399100
C	-0.18787700	-4.30214000	0.12630300
C	4.05282900	-4.31069000	0.69649100
C	2.56156100	-1.82884100	0.67043500
C	4.93837600	-0.48335700	0.51304900
C	4.11383600	0.65915800	0.41830700
C	2.65638300	0.63435700	0.47783600
C	1.91108400	-0.58984800	0.52610300
C	0.50338100	-0.59822500	0.36728600
C	-0.19732800	-1.83308600	0.26955200
C	2.79487800	-6.68342100	0.10530000
C	4.17198800	-6.63497100	0.20835200
C	4.84592100	-5.43006400	0.52208000
C	6.32484000	-5.31294000	0.57571400
C	6.98618100	-4.02764100	0.45266800
C	6.33089100	-2.77103400	0.49603800
C	6.30421800	-0.37000900	0.25444000

C	7.05611600	-1.60802300	0.25250200
C	1.92703300	1.83554700	0.35138100
C	4.75912200	1.92130000	0.17554500
C	6.94430900	0.85545800	-0.01747500
C	8.45010600	-1.59121900	0.00773800
C	8.41764200	-4.01806000	0.27949700
C	7.09588000	-6.45192000	0.65928700
C	-0.20703300	-6.71318700	-0.06481700
C	-1.60095900	-4.28153400	-0.03696700
C	-1.62579500	-1.83451500	0.10031200
C	-0.20803600	0.63977300	0.27928500
C	2.62343200	3.08705500	0.22189000
H	2.03651500	3.99941300	0.16645800
C	3.97154200	3.12343600	0.12223200
H	4.49084300	4.06802700	-0.01557000
C	6.14435800	1.99703300	-0.02264400
C	8.37449700	0.84182300	-0.25887200
C	9.11030000	-2.81016400	0.05730400
H	10.18958400	-2.84924700	-0.07548500
C	9.08536400	-0.31159000	-0.24405700
C	9.14897000	-5.23815000	0.34995400
H	10.22919100	-5.20155600	0.23762000
C	8.50709400	-6.42277100	0.56881300
C	0.51199100	1.81602300	0.29702100
C	-1.64957100	0.60568200	0.14836600
H	-2.18046900	1.55267100	0.10716600
C	-2.31931700	-0.56293800	0.06527800

H	-3.40022000	-0.57635200	-0.04292900
C	-2.28469600	-3.03458200	-0.03649400
H	-3.36511500	-3.03898200	-0.16341000
C	-2.29217700	-5.50117900	-0.20220400
H	-3.37182900	-5.48378300	-0.32076900
C	-1.60256000	-6.69309800	-0.21172600
H	-2.13671800	-7.62971200	-0.33404500
H	10.15648100	-0.29725200	-0.42651200
H	8.87154600	1.78805400	-0.45518900
H	6.59320500	2.97214000	-0.20042100
H	-0.01463700	2.76467100	0.22025600
H	9.06593300	-7.34917600	0.64890400
H	0.29853100	-7.67229300	-0.07227300
H	2.33732500	-7.63608300	-0.13847600
H	4.73146700	-7.54390600	0.01457800
H	6.61423600	-7.41390100	0.79856400
W	4.41226900	-2.38299000	1.37950500
O	4.69384800	-2.18914300	3.05620300
C	7.75021200	-2.79846700	3.38348800
O	7.41769100	-3.83195200	3.70333400

$^{D}TS_{12}$

C	0.54128600	-5.55016100	0.10718100
C	2.00723000	-5.53694500	0.22638000
C	2.66000600	-4.32850000	0.50073900
C	1.91023600	-3.09456300	0.57459200
C	0.51050300	-3.08763000	0.39086500

C	-0.17507100	-4.31699600	0.19102200
C	4.06255900	-4.21978500	0.67397700
C	2.63578600	-1.90616100	0.75383300
C	4.98553400	-0.45604500	0.54154300
C	4.17003300	0.67040000	0.42300100
C	2.71437900	0.60435400	0.52396500
C	1.99038800	-0.63016800	0.61281800
C	0.57358400	-0.62626700	0.50408900
C	-0.16108200	-1.85102300	0.40718200
C	2.83524100	-6.66154500	0.06040400
C	4.20879700	-6.56002900	0.16920600
C	4.87278500	-5.35042800	0.49888900
C	6.36529300	-5.32701300	0.54476600
C	7.09033000	-4.08691900	0.43370300
C	6.43037600	-2.85842900	0.53106700
C	6.36233400	-0.40056500	0.28957800
C	7.11616700	-1.65698600	0.29762700
C	1.98435000	1.81010000	0.41036000
C	4.81605000	1.91939200	0.14764300
C	7.00222000	0.81863900	-0.01743100
C	8.50846700	-1.64079100	0.01813000
C	8.50753200	-4.08489600	0.22297300
C	7.10767100	-6.48848000	0.59297800
C	-0.19032900	-6.71502100	-0.08095300
C	-1.59027600	-4.28803200	0.06498200
C	-1.58930500	-1.84024000	0.27556100
C	-0.13973700	0.61132700	0.43821500

C	2.67685200	3.06170000	0.23220300
H	2.07964200	3.96767500	0.18022900
C	4.02055100	3.11565300	0.08610900
H	4.52281500	4.06384000	-0.08484700
C	6.20366200	1.96621300	-0.05499100
C	8.42863100	0.79490000	-0.27642400
C	9.17889100	-2.86083900	0.01548800
H	10.25316700	-2.88145000	-0.15735100
C	9.13612400	-0.36128700	-0.25536500
C	9.20571100	-5.32460900	0.24462200
H	10.28352100	-5.32818700	0.10718400
C	8.51967800	-6.48785900	0.45987500
C	0.57485000	1.79302100	0.41543900
C	-1.58565900	0.59210000	0.36109900
H	-2.10426700	1.54664700	0.34982400
C	-2.27444200	-0.56569300	0.28643900
H	-3.35841400	-0.56499500	0.21560100
C	-2.26109600	-3.03733500	0.12364000
H	-3.34466100	-3.02786200	0.02608600
C	-2.28508500	-5.50417600	-0.12355900
H	-3.36739200	-5.48760000	-0.21489400
C	-1.59130700	-6.69185300	-0.19098600
H	-2.12621900	-7.62530500	-0.33247400
H	10.20273200	-0.35020900	-0.46352400
H	8.92604300	1.73452000	-0.50113900
H	6.66447500	2.93021700	-0.26116400
H	0.04132900	2.73853600	0.34996000

H	9.04987300	-7.43344800	0.50816100
H	0.31749800	-7.67185400	-0.13744100
H	2.40887800	-7.62621100	-0.19428700
H	4.79227300	-7.44958200	-0.03990300
H	6.61766800	-7.44522400	0.73554000
W	4.61448500	-2.29888800	1.44806800
O	4.92411800	-2.42962200	3.24891200
O	3.13818900	-2.40305600	2.62703400

$^{D}TS_{13}$

C	0.54902000	-5.50605100	0.17796700
C	2.00713900	-5.45745700	0.35315000
C	2.62326700	-4.22368800	0.55282700
C	1.86996900	-2.98337200	0.49428200
C	0.47578100	-3.02562500	0.28775400
C	-0.18512600	-4.28138300	0.15398200
C	4.00996700	-4.07008800	0.75880700
C	2.58684700	-1.80133900	0.62013400
C	4.85974300	-0.31152000	0.47300500
C	4.02667200	0.80635200	0.33924500
C	2.58335500	0.71073000	0.40925100
C	1.90211700	-0.55622400	0.47391100
C	0.48178300	-0.57711400	0.32211500
C	-0.22624000	-1.80755400	0.23742000
C	2.86462100	-6.57805300	0.30660800
C	4.22863400	-6.43576200	0.43623500
C	4.85694400	-5.18095000	0.67476000

C	6.34704500	-5.11073800	0.69653400
C	7.03146200	-3.86454300	0.45607500
C	6.35487600	-2.64146700	0.49489800
C	6.23360500	-0.22881900	0.18019400
C	7.01699200	-1.45830800	0.18264800
C	1.82864500	1.89651900	0.27100900
C	4.65049900	2.06448400	0.04911300
C	6.84305300	0.99653900	-0.15725900
C	8.39826900	-1.42741200	-0.13865500
C	8.44585500	-3.84819500	0.21302800
C	7.12079500	-6.24265900	0.84193800
C	-0.15532200	-6.69558900	0.03998400
C	-1.59660900	-4.28864400	-0.01101800
C	-1.65491600	-1.83570500	0.07709300
C	-0.25883100	0.64640700	0.22915900
C	2.49244200	3.16469500	0.10919300
H	1.87525500	4.05646700	0.04670500
C	3.83615500	3.24653000	-0.01995300
H	4.32347300	4.20255000	-0.19005900
C	6.03003800	2.13403200	-0.17943500
C	8.25946800	0.99538000	-0.46853400
C	9.09272800	-2.63216400	-0.08973200
H	10.16450000	-2.64501100	-0.27682400
C	8.99223800	-0.14500600	-0.46218900
C	9.17498000	-5.06575100	0.32949200
H	10.24815200	-5.05560100	0.15970200
C	8.52938400	-6.21971600	0.67580100

C	0.41985600	1.84635600	0.22935600
C	-1.70123800	0.59482600	0.10692300
H	-2.23713500	1.53892800	0.06314500
C	-2.36556700	-0.57690300	0.03329600
H	-3.44691200	-0.59943000	-0.06881400
C	-2.29620600	-3.04926300	-0.03772200
H	-3.37699000	-3.06992300	-0.16104300
C	-2.26364100	-5.52507800	-0.14554900
H	-3.34293900	-5.53361900	-0.26902200
C	-1.54976300	-6.70408400	-0.11918300
H	-2.06613300	-7.65317000	-0.22047000
H	10.05011800	-0.11572700	-0.70874000
H	8.72710000	1.94290900	-0.72227500
H	6.47194000	3.10299600	-0.40313100
H	-0.13548400	2.77756600	0.14571400
H	9.08691000	-7.14214900	0.80080300
H	0.37044800	-7.64413300	0.06273400
H	2.46405400	-7.56971500	0.12361600
H	4.84005200	-7.32177100	0.30958400
H	6.65854300	-7.19250300	1.08636700
W	4.52704100	-2.11463900	1.44646800
O	5.59541100	-2.48920300	3.18047500
O	3.97982900	-1.90431200	3.17297900
C	6.98927700	-3.41297100	3.46450600
O	7.23112200	-4.21294400	4.23671700

$^{D}TS_{15}$

C	0.53853200	-5.46164900	0.09520400
C	1.99195100	-5.47134500	0.30658000
C	2.65013400	-4.27315600	0.59756900
C	1.93823800	-3.00660400	0.57964100
C	0.54417600	-2.99160500	0.33550300
C	-0.15528300	-4.21546100	0.12493900
C	4.04498300	-4.19637900	0.82657400
C	2.66918100	-1.84297600	0.79369000
C	4.95080700	-0.46093300	0.43645600
C	4.17612300	0.69934600	0.40268900
C	2.74252400	0.66623600	0.58113600
C	2.02612700	-0.57699100	0.65793000
C	0.60752600	-0.55133400	0.50858800
C	-0.12969800	-1.75692300	0.35773300
C	2.79449000	-6.62646500	0.21169600
C	4.16200600	-6.55335200	0.35670300
C	4.83423900	-5.34052300	0.65855600
C	6.32047500	-5.32227400	0.64474100
C	7.02136500	-4.09500100	0.38057400
C	6.36035600	-2.85694900	0.39093300
C	6.31431700	-0.42598600	0.09736400
C	7.05490200	-1.68137700	0.07841000
C	2.02794800	1.87984200	0.50374800
C	4.83854900	1.93978300	0.11836600
C	6.96936400	0.78477900	-0.20842100
C	8.43678600	-1.68594400	-0.23588000
C	8.43188300	-4.11141600	0.12190400

C	7.06846500	-6.47412400	0.76672700
C	-0.19858700	-6.61985300	-0.11852500
C	-1.56543000	-4.17304400	-0.05160600
C	-1.55788000	-1.73419900	0.19147900
C	-0.09592800	0.69737200	0.47274800
C	2.73511800	3.12481300	0.33761000
H	2.15369000	4.04248000	0.33472800
C	4.07203000	3.15630600	0.13569200
H	4.58952500	4.09740400	-0.02818300
C	6.20655500	1.95754700	-0.17130100
C	8.37913300	0.74071300	-0.53993800
C	9.09834600	-2.91150100	-0.19603800
H	10.16685200	-2.94973600	-0.39924200
C	9.07311200	-0.42351100	-0.55276200
C	9.13842500	-5.34351900	0.22354000
H	10.21010800	-5.35264700	0.04522500
C	8.47285100	-6.48597300	0.57031900
C	0.61799600	1.87673900	0.51108900
C	-1.53945100	0.69198500	0.35785000
H	-2.04965500	1.65113200	0.36653400
C	-2.23416300	-0.45623000	0.22289700
H	-3.31591000	-0.44500000	0.12324900
C	-2.23228500	-2.91769700	-0.00600800
H	-3.31196200	-2.90068600	-0.13852200
C	-2.26744700	-5.37901800	-0.26201600
H	-3.34541800	-5.34716200	-0.39291200
C	-1.58986100	-6.57848400	-0.29542100

H	-2.13241900	-7.50472300	-0.45456500
H	10.12999700	-0.42906800	-0.80463500
H	8.87650300	1.67547700	-0.78410600
H	6.68370300	2.91255800	-0.38150800
H	0.08950600	2.82639800	0.47499300
H	9.01117100	-7.42153000	0.68064700
H	0.29663100	-7.58436400	-0.13953700
H	2.35049500	-7.58638200	-0.02864700
H	4.73998600	-7.45465200	0.18463800
H	6.58337600	-7.41063400	1.02053800
W	4.68375200	-2.25060400	1.47183600
O	5.45222400	-1.89788700	3.22356200
O	4.15697200	-1.25782400	3.40180200
C	3.30358300	-2.59555500	3.05808000
O	2.45951000	-2.95388600	3.77182400

$^{D}TS_{23}$

C	0.44020600	-5.58418100	0.15796900
C	1.88456900	-5.57300100	0.43359100
C	2.51642000	-4.36347500	0.72076900
C	1.79616800	-3.10444800	0.59089600
C	0.41913700	-3.10551600	0.27868900
C	-0.26180500	-4.34204500	0.08948400
C	3.89245500	-4.24248800	1.03045700
C	2.51513300	-1.93197900	0.76187900
C	4.88893000	-0.45949300	0.87666400
C	4.06598600	0.66405800	0.68510100

C	2.61366200	0.58176200	0.62136500
C	1.88603100	-0.66522300	0.59408200
C	0.48329600	-0.65204100	0.33057800
C	-0.24239200	-1.86809200	0.17861800
C	2.72080400	-6.70582500	0.39149000
C	4.08408400	-6.58607100	0.55579100
C	4.73013000	-5.35510200	0.86362900
C	6.23231200	-5.30326800	0.79274300
C	6.96092900	-4.07732900	0.66032700
C	6.41363000	-2.81780700	1.07232900
C	6.28388000	-0.38563100	0.68479400
C	7.06322800	-1.60340200	0.71302000
C	1.88974200	1.78547700	0.44352600
C	4.71814500	1.91891700	0.45480700
C	6.91040900	0.83801500	0.34091400
C	8.37031500	-1.61618600	0.15745000
C	8.31093000	-4.07425500	0.20167000
C	6.97115100	-6.47212200	0.66045900
C	-0.28098000	-6.75446400	-0.03787200
C	-1.65640600	-4.31252200	-0.18195900
C	-1.65523700	-1.85904600	-0.09008600
C	-0.21905700	0.58816100	0.19859700
C	2.57918100	3.04548400	0.36638400
H	1.98005500	3.94694100	0.27381200
C	3.93028100	3.11197600	0.34416400
H	4.44149800	4.06350600	0.22867500
C	6.11125500	1.97688300	0.29307900

C	8.31305300	0.82039400	-0.02165000
C	8.98527100	-2.84497600	-0.02362700
H	10.01718800	-2.88159000	-0.36642600
C	8.99359500	-0.34255500	-0.14529500
C	8.98342700	-5.30784600	0.01370200
H	10.01439900	-5.30368200	-0.32821800
C	8.33259600	-6.48007000	0.29524800
C	0.48858300	1.76836500	0.27982000
C	-1.64716100	0.57333500	-0.04201600
H	-2.15642900	1.52999500	-0.11812200
C	-2.32919700	-0.58230200	-0.18053500
H	-3.39921900	-0.57832100	-0.36846300
C	-2.31828100	-3.05499600	-0.25723400
H	-3.38687400	-3.04606300	-0.46146100
C	-2.34196200	-5.53195500	-0.37219800
H	-3.40870200	-5.51347100	-0.57673400
C	-1.66060200	-6.72755400	-0.29848400
H	-2.19096000	-7.66346800	-0.44167700
H	10.02509800	-0.34543800	-0.48568800
H	8.79364500	1.76614700	-0.25571200
H	6.56686400	2.94014700	0.07342300
H	-0.03432700	2.71543700	0.16954300
H	8.84648600	-7.43077900	0.19970200
H	0.21820700	-7.71589300	0.01898800
H	2.31327000	-7.68491700	0.16206900
H	4.67041200	-7.48318500	0.40284800
H	6.49987600	-7.43399800	0.81796100

W	4.32043300	-2.24688800	1.77647500
O	6.09797500	-3.00903900	2.64949000
O	3.76289900	-1.74685400	3.31805600
C	6.83714600	-2.34764900	3.70440000
O	7.67975100	-1.57346400	3.44097900

$^{D}TS_{24}$

C	0.57555900	-5.45982900	0.52408200
C	2.02759400	-5.44079700	0.77833400
C	2.69025400	-4.23541200	1.10444300
C	1.89261500	-3.01452900	1.08827800
C	0.56205800	-2.99659900	0.58072400
C	-0.11400700	-4.22520800	0.35411100
C	4.12075400	-4.18838900	1.22770900
C	2.40891700	-1.79859100	1.49311800
C	5.08109300	-0.58803300	0.79113700
C	4.24303800	0.57575700	0.81451600
C	2.77746800	0.61118100	0.94829500
C	1.93619300	-0.55361500	1.03203400
C	0.59592500	-0.54435700	0.58541100
C	-0.10574800	-1.76757900	0.40842300
C	2.80826400	-6.58820300	0.58367200
C	4.18486600	-6.53382700	0.62916100
C	4.87814300	-5.34324800	0.91845300
C	6.35385800	-5.34709600	0.70613700
C	7.03350800	-4.14886500	0.29819500
C	6.39026700	-2.89948200	0.35734500

C	6.37914300	-0.48327500	0.25874000
C	7.07983000	-1.72942700	0.03992800
C	2.08968500	1.83106200	0.71607500
C	4.89806800	1.82079000	0.49847300
C	7.00944100	0.73458000	-0.06698600
C	8.42308400	-1.75046500	-0.42278200
C	8.40682000	-4.18057200	-0.11237800
C	7.09662200	-6.51033400	0.77227700
C	-0.14917300	-6.63715600	0.38742600
C	-1.49155700	-4.19999000	-0.00305600
C	-1.48346100	-1.75413800	0.00548100
C	-0.05154700	0.70428900	0.34286100
C	2.81558000	3.06239800	0.60301000
H	2.25429500	3.98969900	0.53423100
C	4.16192100	3.05396200	0.50195200
H	4.71489100	3.97773500	0.35675000
C	6.25026200	1.88132300	0.11519900
C	8.37318800	0.69500200	-0.55961900
C	9.05554800	-2.98335100	-0.50063800
H	10.09648400	-3.03642400	-0.81127800
C	9.04059700	-0.47330100	-0.72569700
C	9.09786400	-5.41956500	-0.05490700
H	10.14168300	-5.45534800	-0.35372300
C	8.46002700	-6.54422500	0.40246500
C	0.69105200	1.85704600	0.48765900
C	-1.44901700	0.69176900	-0.03635500
H	-1.94298100	1.64560100	-0.19908100

C	-2.12364100	-0.46827800	-0.19199700
H	-3.17069900	-0.46212000	-0.48220900
C	-2.14313800	-2.94808500	-0.18151700
H	-3.19031300	-2.94453900	-0.47505200
C	-2.17369500	-5.42682500	-0.15404300
H	-3.22681800	-5.41646000	-0.42029700
C	-1.51292000	-6.61757300	0.05416500
H	-2.04692000	-7.55755400	-0.04191300
H	10.06406000	-0.47094100	-1.09012400
H	8.85832900	1.63803800	-0.79634400
H	6.68277400	2.85771400	-0.09219600
H	0.21665300	2.82293600	0.33040500
H	9.00170000	-7.48134900	0.47977000
H	0.32904900	-7.59657400	0.54978700
H	2.33865400	-7.52160200	0.29501900
H	4.74268000	-7.42007000	0.34829000
H	6.63901900	-7.42563500	1.13255400
W	5.09318400	-2.41850800	1.85268100
O	6.48902700	-2.96785700	3.09438100
O	3.40368800	-1.79630100	2.39897800
C	7.82376400	-3.10645100	2.91462200
O	8.64633700	-2.29131300	2.70616400

$^{D}TS_{53}$

C	0.21305100	-5.55262600	0.33121900
C	1.66523800	-5.54343800	0.55019800
C	2.29224100	-4.33450700	0.84664000

C	1.57176700	-3.07457600	0.81314600
C	0.18299100	-3.07694100	0.56528100
C	-0.49620800	-4.31298900	0.35397000
C	3.66428300	-4.21844400	1.12694900
C	2.30271200	-1.90463300	0.99749700
C	4.63570700	-0.47406700	0.89497700
C	3.81550500	0.66182900	0.78471800
C	2.36741100	0.60511200	0.84130300
C	1.65232700	-0.64064600	0.87109700
C	0.23520400	-0.63170100	0.69470400
C	-0.49369100	-1.84416600	0.54867300
C	2.51110300	-6.66939900	0.46129400
C	3.87344800	-6.55325900	0.64767700
C	4.50049000	-5.32809800	1.00497600
C	5.98452200	-5.25224800	1.10889100
C	6.68885100	-3.99927500	0.96420600
C	6.04945400	-2.74281400	0.98825900
C	6.01043400	-0.36395100	0.65084600
C	6.77118200	-1.59386500	0.69444800
C	1.63581000	1.80926600	0.72993600
C	4.46054000	1.92278800	0.53861100
C	6.64710800	0.85479600	0.35740000
C	8.16272100	-1.59043100	0.43669200
C	8.11466900	-4.01073100	0.77359800
C	6.73550600	-6.40254200	1.23211000
C	-0.50976100	-6.71825300	0.11277000
C	-1.90270200	-4.28388100	0.15359100

C	-1.91855600	-1.83653700	0.35523400
C	-0.47876600	0.60811900	0.62810200
C	2.32291700	3.06761700	0.60818700
H	1.72489700	3.97329900	0.56531000
C	3.66835800	3.12087400	0.49326700
H	4.17878700	4.07004600	0.35539300
C	5.84343400	1.99637100	0.33575400
C	8.07768000	0.83618800	0.11380800
C	8.81553300	-2.81362700	0.51248600
H	9.89321100	-2.86428600	0.37135100
C	8.79221100	-0.31556000	0.14793500
C	8.82019900	-5.24330700	0.87009000
H	9.89918200	-5.23938300	0.74199000
C	8.14780200	-6.40231500	1.13525900
C	0.22698000	1.79098600	0.67507300
C	-1.91927700	0.59088100	0.47974000
H	-2.43522200	1.54677200	0.46178100
C	-2.60459500	-0.56349600	0.34887100
H	-3.68387000	-0.56096500	0.22549100
C	-2.57830200	-3.03150500	0.17065900
H	-3.65595000	-3.02541800	0.02109500
C	-2.59007000	-5.49794300	-0.06130000
H	-3.66572700	-5.47752100	-0.21172800
C	-1.89988700	-6.69081100	-0.08017300
H	-2.43129800	-7.62266600	-0.24376900
H	9.86162400	-0.30103800	-0.04381300
H	8.57189800	1.77872000	-0.10542600

H	6.29151000	2.96991600	0.14784800
H	-0.30591500	2.73685200	0.61364300
H	8.68521800	-7.33887300	1.24147600
H	-0.00139100	-7.67647800	0.09882300
H	2.10604100	-7.64150800	0.19973500
H	4.48011700	-7.43697500	0.48435000
H	6.24209200	-7.35231800	1.40565100
W	4.16953400	-2.27936100	1.87978300
O	5.14565100	-3.74436900	3.47141000
O	3.96021800	-1.74712700	3.58253400
C	4.81484100	-3.08954100	4.45180100
O	4.78923000	-2.92851300	5.60667600

$^{D}TS_{60}$

C	0.50600100	-5.58768400	0.11078200
C	1.97090900	-5.58722600	0.21255400
C	2.65459300	-4.38189600	0.41623700
C	1.91823400	-3.10088700	0.44688400
C	0.50743900	-3.10836800	0.27553900
C	-0.19231800	-4.34308300	0.13525200
C	4.07552600	-4.36473200	0.51507200
C	2.60156100	-1.88482800	0.55892500
C	4.94969600	-0.59113800	0.49648600
C	4.14114100	0.57391800	0.41571900
C	2.68908500	0.57120100	0.42828300
C	1.94244400	-0.64767300	0.44332500
C	0.53115900	-0.64607400	0.31649700

C	-0.17960600	-1.87388800	0.24231400
C	2.76313700	-6.74917100	0.07120600
C	4.14091300	-6.71561300	0.12566100
C	4.84357800	-5.50753200	0.35901700
C	6.31708600	-5.40924400	0.38740600
C	6.97651400	-4.12304200	0.35844800
C	6.31573300	-2.86159500	0.44911600
C	6.33234000	-0.45730200	0.35226000
C	7.07811200	-1.69525400	0.34024300
C	1.96859000	1.78148400	0.33069900
C	4.80362100	1.83932100	0.26148400
C	6.98976100	0.77736400	0.15769000
C	8.48169100	-1.67870300	0.12769100
C	8.41149000	-4.11222500	0.21415200
C	7.08113200	-6.56164000	0.37653000
C	-0.23478700	-6.75704500	-0.01540800
C	-1.60921300	-4.31420400	0.01025500
C	-1.61282300	-1.86632700	0.11209500
C	-0.17345100	0.59847000	0.25123200
C	2.67640500	3.02640700	0.25643800
H	2.10222300	3.94726100	0.20958400
C	4.02948300	3.04762300	0.21287000
H	4.56411200	3.99004000	0.12904900
C	6.20032100	1.91860500	0.13980800
C	8.43169900	0.76577400	-0.00234900
C	9.12579200	-2.89838300	0.09078900
H	10.20558200	-2.94319500	-0.03352300

C	9.13456500	-0.39083200	-0.01656800
C	9.13379100	-5.33397000	0.19290700
H	10.21572700	-5.29201200	0.10107900
C	8.48617500	-6.53488800	0.29496500
C	0.55305000	1.76963400	0.27359300
C	-1.61729000	0.57413000	0.14801700
H	-2.14211800	1.52488600	0.11595700
C	-2.29726800	-0.58955900	0.08315000
H	-3.38032000	-0.59560100	-0.00132000
C	-2.28434800	-3.06202600	0.00974800
H	-3.36779600	-3.06011500	-0.08732900
C	-2.31295900	-5.53037300	-0.11439300
H	-3.39539300	-5.50684100	-0.20256700
C	-1.63250300	-6.72859900	-0.12249300
H	-2.17705500	-7.66272500	-0.21414400
H	10.21291000	-0.37599400	-0.14989300
H	8.94132600	1.71784800	-0.12401900
H	6.65990900	2.89694500	0.01716400
H	0.03122000	2.72234900	0.21616000
H	9.04192700	-7.46654100	0.29706500
H	0.26295200	-7.72005800	-0.02396900
H	2.28602400	-7.70462100	-0.11690400
H	4.67677000	-7.64384900	-0.03653200
H	6.59542500	-7.52894100	0.43512600
W	4.41983600	-2.48959400	1.27889200
O	5.35982500	-1.97756700	2.84545100
C	6.58922100	-1.37153700	3.12991800

O	7.09734500	-1.21142300	4.16733500
